# A statistical measure for the skewness of X chromosome inactivation based on case-control design

**DOI:** 10.1186/s12859-018-2587-2

**Published:** 2019-01-07

**Authors:** Peng Wang, Yu Zhang, Bei-Qi Wang, Jian-Long Li, Yi-Xin Wang, Dongdong Pan, Xian-Bo Wu, Wing Kam Fung, Ji-Yuan Zhou

**Affiliations:** 1 State Key Laboratory of Organ Failure Research, Ministry of Education, and Guangdong Provincial Key Laboratory of Tropical Disease Research, Department of Biostatistics, School of Public Health, Southern Medical University, No. 1023, South Shatai Road, Baiyun District, Guangzhou, 510515 China; 2 Department of Occupational and Environmental Health, School of Public Health, Tongji Medical College, Huazhong University of Science and Technology, Wuhan, China; 3 Yunnan Key Laboratory of Statistical Modeling and Data Analysis, Yunnan University, Kunming, China; 4 Department of Epidemiology, School of Public Health, Southern Medical University, Guangzhou, China; 5 Department of Statistics and Actuarial Science, University of Hong Kong, Hong Kong, China

**Keywords:** X chromosome inactivation, Skewness, Case-control design, Confidence interval, Graves’ disease

## Abstract

**Background:**

Skewed X chromosome inactivation (XCI), which is a non-random process, is frequently observed in both healthy and affected females. Furthermore, skewed XCI has been reported to be related to many X-linked diseases. However, no statistical method is available in the literature to measure the degree of the skewness of XCI for case-control design. Therefore, it is necessary to develop methods for such a task.

**Results:**

In this article, we first proposed a statistical measure for the degree of XCI skewing by using a case-control design, which is a ratio of two logistic regression coefficients after a simple reparameterization. Based on the point estimate of the ratio, we further developed three types of confidence intervals (the likelihood ratio, Fieller’s and delta methods) to evaluate its variation. Simulation results demonstrated that the likelihood ratio method and the Fieller’s method have more accurate coverage probability and more balanced tail errors than the delta method. We also applied these proposed methods to analyze the Graves’ disease data for their practical use and found that rs3827440 probably undergoes a skewed XCI pattern with 68.7*%* of cells in heterozygous females having the risk allele *T* active, while the other 31.3*%* of cells keeping the normal allele *C* active.

**Conclusions:**

For practical application, we suggest using the Fieller’s method in large samples due to the non-iterative computation procedure and using the LR method otherwise for its robustness despite its slightly heavy computational burden.

**Electronic supplementary material:**

The online version of this article (10.1186/s12859-018-2587-2) contains supplementary material, which is available to authorized users.

## Background

X chromosome inactivation (XCI) is an epigenetic phenomenon. Under XCI, one of two X chromosomes in females is silenced during early embryonic development to achieve dosage compensation between two sexes [1]. As such, the genetic effect of two risk alleles in females is expected to be equivalent to that of one risk allele in males. Most of X-linked genes undergo XCI and only about 15% of genes on X chromosome escape from XCI (XCI-E) [2]. Both alleles in the genes under XCI-E will be active, which are similar to autosomal genes. Generally, XCI has been treated as random (XCI-R) where both maternal and paternal X chromosomes have equal chance to be inactivated, i.e. for an X-linked gene, nearly 50% of the cells have one allele active while the remaining cells have the other allele active. However, recent studies have revealed that the skewed XCI (XCI-S) is a biological plausibility and even a common feature in both healthy and affected females [3–5]. XCI-S is a non-random process, which has been defined as a significant deviation from XCI-R, for instance, the inactivation of one of the alleles in more than 75% of cells [6–8].

The mechanism of XCI-S remains mysterious and XCI-S in human may be likely caused by secondary selection [6, 9, 10]. Specifically, the initial choice of active X chromosome is considered as random. However, during body growth, when an X-linked mutation affects cells proliferation or survival, there will be a larger or smaller proportion of cells with an active mutant allele. Due to the selection pressure, this type of secondary skewing varies in different tissues and is also associated with age. For heterozygous females, positive selection cells with mutant allele will lead to more severe expression of the disease, whereas negative selection cells with mutant allele can provide protection from deleterious effects [11, 12]. For example, in heterozygous females with a mutant FoxP3 allele, the XCI-S against the mutant allele in specific tissues can prevent autoimmune disease, whereas the XCI-S towards the mutant allele in breast epithelial cells can result in breast cancer [13]. On the other hand, some diseases, such as ovarian cancer, Rett syndrome, Klinefelter syndrome, and recurrent miscarriages, are reported to be related to XCI-S [14–17]. Therefore, it is necessary to develop methods for measuring such XCI skewing.

Recently, there has been an increasing interest to incorporate the information on XCI into X-chromosome genetic association studies [18–23]. Clayton’s method first takes XCI-R into account and treats males as homozygous females [18]. In this regard, two genotypes of males are coded as 0 or 2, while three genotypes of females are coded as 0, 1 or 2, respectively. This coding strategy also implies that the genetic effect of heterozygous genotype in females lies midway between two homozygous genotypes, which seems reasonable as in heterozygous females about half of cells express the mutant allele while the rest of cells express the normal allele. However, this method does not consider the XCI-E and XCI-S patterns. So, a resampling-based method was proposed by maximizing the likelihood ratio (LR) over all the three biological patterns (XCI-E, XCI-R and XCI-S), where the three genotypes of females are coded as 0, *γ* or 2 under XCI-S [21]. Note that *γ* is an unknown parameter which is used to measure the degree of XCI skewing. For instance, *γ*=1 represents XCI-R; *γ*=1.5 indicates XCI-S where 75% of the cells have the mutant allele active, whereas the other 25% of the cells have the normal allele active. On the other hand, the detection of XCI-S is either by measuring the level of methylation or by integrative analysis of whole exome and RNA sequencing data [24, 25]. Although Xu et al. has recently developed a statistical measure for the skewness of XCI based on family trios [26], there is still no statistical method available in the literature to measure the skewness of XCI for case-control design.

Therefore, in this article, we first showed that *γ* can be represented as a ratio of two logistic regression coefficients after a simple reparameterization, based on case-control data. We then obtained the point estimate of *γ* by the maximum likelihood estimates (MLEs) of these two regression coefficients. Further, we derived the confidence interval (CI) of *γ* by the delta method, the Fieller’s method and the LR method. We also applied all the proposed approaches to analyze the Graves’ disease data for their practical use.

## Results

### Statistical properties of confidence interval

Tables 1 and 2 list the estimated coverage probability (CP), left tail error (ML), right tail error (MR) (missing the true value of *γ*), ML/(ML+MR) and proportion of the discontinuous CIs (DP) of the LR, Fieller’s and delta methods under various simulation settings with *N*=500, *ρ*=0, and *λ*_2_=1.5 and 2, respectively. From the tables, the LR and Fieller’s methods control the CP well when *p*=0.3. However, when *p*=0.1, both the LR and Fieller’s methods appear to overestimate the CP except for *γ*=0. Note that the CPs of the LR method are closer to the pre-set level than the Fieller’s method, which indicates the robustness property of the LR method for relatively small samples. Besides, the delta method generally has the worst CP under all the situations. When *p*=0.1, the delta method overestimates the CP for *γ*=0, while underestimates the CP for *γ*=1, 1.5 and 2, irrespective of *λ*_2_ being 1.5 or 2. When *p* increases from 0.1 to 0.3, the delta method overestimates the CP for *γ*=0, 0.5 and 1, while underestimates the CP for *γ*=1.5 and 2, regardless of *λ*_2_=1.5 or 2. From the estimated ML, MR and ML/(ML+MR) values, we find that ML and MR of the delta-type CIs are not balanced since nearly all the values of ML/(ML+MR) are far away from 0.5, while the LR and Fieller’s methods have more balanced ML and MR than the delta method, especially when *p*=0.3. On the other hand, we see that the values of the DP for both the LR and Fieller’s methods are not over 3% under our simulation settings. Further, when *p* increases from 0.1 to 0.3 or *λ*_2_ changes from 1.5 to 2, DP will generally become smaller.

**Table 1:** Estimated CP (%), ML (%), MR (%), ML/(ML+MR) and DP (%) of the two-sided 95% CI when *N*=500, *ρ*=0 and *λ*_2_=1.5 for the LR, Fieller’s and delta methods

		LR				Fieller				Delta			
*p*	*γ*	CP	(ML, MR)	$\frac {\text {ML}}{\text {ML+MR}}$	DP	CP	(ML, MR)	$\frac {\text {ML}}{\text {ML+MR}}$	DP	CP	(ML, MR)	$\frac {\text {ML}}{\text {ML+MR}}$	DP
0.1	0	94.75	(4.78, 0.47)	0.91	1.96	94.93	(4.89, 0.18)	0.96	2.28	100	(0, 0)	—	0
	0.5	95.78	(1.49, 1.65)	0.47	1.44	96.81	(1.58, 0.62)	0.72	1.49	95.72	(0, 4.28)	0	0
	1	96.13	(0.90, 2.33)	0.28	1.82	98.18	(0.73, 0.60)	0.55	1.82	79.38	(0, 20.62)	0	0
	1.5	96.39	(0.64, 2.84)	0.18	1.69	98.72	(0.37, 0.75)	0.33	1.85	70.83	(0, 29.17)	0	0
	2	96.68	(0, 3.32)	0	2.35	98.99	(0, 1.01)	0	2.49	67.71	(0, 32.29)	0	0
0.3	0	94.72	(3.88, 1.40)	0.73	1.27	94.75	(3.87, 1.38)	0.74	1.31	99.22	(0.78, 0)	1	0
	0.5	95.21	(2.45, 1.96)	0.56	0.73	95.25	(2.44, 1.93)	0.56	0.73	99.75	(0.02, 0.23)	0.08	0
	1	94.77	(1.99, 2.85)	0.41	0.69	94.88	(1.98, 2.78)	0.42	0.65	96.70	(0, 3.30)	0	0
	1.5	95.22	(1.26, 3.33)	0.27	0.88	95.39	(1.25, 3.14)	0.28	0.91	92.29	(0, 7.71)	0	0
	2	94.72	(0.37, 4.91)	0.07	1.29	95.16	(0.34, 4.50)	0.07	1.36	88.11	(0, 11.89)	0	0

**Table 2:** Estimated CP (%), ML (%), MR (%), ML/(ML+MR) and DP (%) of the two-sided 95% CI when *N*=500, *ρ*=0 and *λ*_2_=2 for the LR, Fieller’s and delta methods

		LR				Fieller				Delta			
*p*	*γ*	CP	(ML, MR)	$\frac {\text {ML}}{\text {ML+MR}}$	DP	CP	(ML, MR)	$\frac {\text {ML}}{\text {ML+MR}}$	DP	CP	(ML, MR)	$\frac {\text {ML}}{\text {ML+MR}}$	DP
0.1	0	94.59	(4.59, 0.82)	0.85	1.97	94.78	(4.71, 0.51)	0.90	2.72	100	(0, 0)	—	0
	0.5	95.24	(2.12, 1.96)	0.52	1.10	96.20	(2.17, 0.86)	0.72	1.28	94.44	(0, 5.56)	0	0
	1	96.07	(1.39, 2.38)	0.37	1.14	97.78	(1.27, 0.73)	0.64	1.23	84.05	(0, 15.95)	0	0
	1.5	96.56	(0.99, 2.39)	0.29	0.90	98.37	(0.85, 0.72)	0.54	0.98	81.08	(0, 18.92)	0	0
	2	96.49	(0.01, 3.50)	0	0.43	98.37	(0.01, 1.62)	0.01	0.43	79.71	(0, 20.29)	0	0
0.3	0	94.88	(2.80, 2.32)	0.55	0.60	94.93	(2.79, 2.28)	0.55	0.61	98.10	(1.89, 0.01)	0.99	0
	0.5	95.01	(2.40, 2.54)	0.49	0.14	95.01	(2.40, 2.53)	0.49	0.15	99.13	(0.20, 0.67)	0.23	0
	1	95.15	(2.10, 2.71)	0.44	0.08	95.28	(2.09, 2.60)	0.45	0.09	96.37	(0, 3.63)	0	0
	1.5	94.81	(2.03, 3.14)	0.39	0.21	95.03	(2.05, 2.90)	0.41	0.22	92.51	(0, 7.49)	0	0
	2	94.88	(1.80, 3.32)	0.35	0.08	95.18	(1.78, 3.04)	0.37	0.08	91.80	(0, 8.20)	0	0

Tables 3 and 4 give the estimated CP, ML, MR, ML/(ML+MR) and DP of the LR, Fieller’s and delta methods when *N*=2000, *ρ*=0, and *λ*_2_=1.5 and 2, respectively. When *N* increases from 500 to 2000, the LR method has similar performance with the Fieller’s method. It can be seen from the tables that the CPs of all the methods are more accurate. Both the LR and Fieller’s methods control the CP well, while the delta method still generally has the poor CP, especially when *p*=0.1. Note that when *p*=0.3, all the values of ML/(ML+MR) for the LR method and the Fieller’s method are around 0.5 regardless of *λ*_2_ being 1.5 or 2. But when *p*=0.1, the values of ML/(ML+MR) for the LR method and the Fieller’s method are deviated from 0.5, especially when *γ*_0_ are 0 and 2. Further, the Fieller’s method has slightly more balanced tail errors than the LR method when *p*=0.1. In addition, the delta method has the most unbalanced tail errors. We also find that the values of the DP generally decrease to be less than 2% when *N* increases to be 2000. In general, the LR method and the Fieller’s method control the CP well with the relatively balanced tail errors on the left and on the right. All the other results of CP, ML, MR, ML/(ML+MR) and DP with *ρ*=0.05 are given in Tables S1-S4 [see Additional file 1], which are similar to those in Tables 1, 2, 3 and 4 except for *N*=500 and *p*=0.1, indicating that Hardy-Weinberg disequilibrium has limited effect on the results. Notice that under the scenario of *N*=500 and *p*=0.1, we observe that the CPs of all the methods in Additional file 1: Tables S1 and S2 are better than those in Tables 1 and 2, respectively. One possible explanation of this phenomenon is that the genotype frequency of *AA* in the control sample increases from 0.01 to 0.0145 when *ρ* changes from 0 to 0.05.

**Table 3:** Estimated CP (%), ML (%), MR (%), ML/(ML+MR) and DP (%) of the two-sided 95% CI when *N*=2000, *ρ*=0 and *λ*_2_=1.5 for the LR, Fieller’s and delta methods

		LR				Fieller				Delta			
*p*	*γ*	CP	(ML, MR)	$\frac {\text {ML}}{\text {ML+MR}}$	DP	CP	(ML, MR)	$\frac {\text {ML}}{\text {ML+MR}}$	DP	CP	(ML, MR)	$\frac {\text {ML}}{\text {ML+MR}}$	DP
0.1	0	94.89	(4.31, 0.80)	0.84	2.04	94.91	(4.36, 0.73)	0.86	2.04	99.98	(0.01, 0.01)	0.50	0
	0.5	94.38	(2.10, 2.84)	0.43	1.03	94.78	(2.13, 2.43)	0.47	0.96	93.29	(0, 6.71)	0	0
	1	95.09	(1.53, 3.19)	0.32	0.73	95.88	(1.52, 2.43)	0.38	0.71	84.16	(0, 15.84)	0	0
	1.5	94.49	(1.24, 4.21)	0.23	0.56	95.59	(1.25, 3.13)	0.29	0.56	81.18	(0, 18.82)	0	0
	2	94.68	(0.01, 5.31)	0	0.22	95.81	(0.01, 4.18)	0	0.22	81.01	(0, 18.99)	0	0
0.3	0	95.23	(2.59, 2.18)	0.54	0.35	95.23	(2.59, 2.18)	0.54	0.33	97.89	(2.07, 0.04)	0.98	0
	0.5	95.00	(2.66, 2.31)	0.54	0.07	95.01	(2.66, 2.30)	0.54	0.07	99.08	(0.12, 0.80)	0.13	0
	1	94.80	(2.60, 2.56)	0.50	0.07	94.86	(2.60, 2.51)	0.51	0.07	95.91	(0, 4.09)	0	0
	1.5	95.05	(2.16, 2.79)	0.44	0.03	95.10	(2.16, 2.74)	0.44	0.03	93.58	(0, 6.42)	0	0
	2	94.58	(2.34, 3.08)	0.43	0.01	94.68	(2.34, 2.98)	0.44	0.01	91.86	(0, 8.14)	0	0

**Table 4:** Estimated CP (%), ML (%), MR (%), ML/(ML+MR) and DP (%) of the two-sided 95% CI when *N*=2000, *ρ*=0 and *λ*_2_=2 for the LR, Fieller’s and delta methods

		LR				Fieller				Delta			
*p*	*γ*	CP	(ML, MR)	$\frac {\text {ML}}{\text {ML+MR}}$	DP	CP	(ML, MR)	$\frac {\text {ML}}{\text {ML+MR}}$	DP	CP	(ML, MR)	$\frac {\text {ML}}{\text {ML+MR}}$	DP
0.1	0	95.45	(2.89, 1.66)	0.64	1.57	95.48	(2.89, 1.63)	0.64	1.66	99.81	(0.14, 0.05)	0.74	0
	0.5	94.92	(2.24, 2.75)	0.45	0.18	95.27	(2.28, 2.34)	0.49	0.23	92.63	(0, 7.37)	0	0
	1	94.39	(2.38, 3.21)	0.43	0.12	95.31	(2.39, 2.28)	0.51	0.11	88.87	(0, 11.13)	0	0
	1.5	94.77	(1.56, 3.67)	0.30	0	95.77	(1.61, 2.62)	0.38	0	87.92	(0, 12.08)	0	0
	2	94.45	(0.42, 5.13)	0.08	0	95.57	(0.42, 4.01)	0.09	0	87.32	(0, 12.68)	0	0
0.3	0	95.02	(2.68, 2.30)	0.54	0.01	95.03	(2.67, 2.30)	0.54	0.01	96.64	(3.08, 0.28)	0.92	0
	0.5	94.97	(2.37, 2.66)	0.47	0	94.97	(2.37, 2.66)	0.47	0	96.94	(1.04, 2.02)	0.34	0
	1	95.13	(2.40, 2.47)	0.49	0	95.17	(2.40, 2.43)	0.50	0	96.05	(0.16, 3.79)	0.04	0
	1.5	94.68	(2.78, 2.54)	0.52	0	94.76	(2.79, 2.45)	0.53	0	94.65	(0, 5.35)	0	0
	2	94.86	(2.53, 2.61)	0.49	0	94.89	(2.56, 2.55)	0.50	0	93.81	(0, 6.19)	0	0

### Sizes and powers

We also simulated the corresponding size and power for testing *γ*=*γ*_0_ [see Appendix B of Additional file 1]. The size results are given in Additional file 1: Tables S5–S8 and the power results are displayed in Figures S1-S12 [see Additional file 1]. It can be seen that the LR method and the Fieller’s method control the size well except for *N*=500 and *p*=0.1, while the size of the delta method is either conservative or inflated. On the other hand, the power of the LR method and the Fieller’s method are close to each other, but the LR method is generally slightly more powerful than the Fieller’s method. However, the power of the delta method can be quite different from those of the LR and Fieller’s methods.

### Application to Graves’ disease data

The GPR174 gene is located on X chromosome, which is associated with autoimmune thyroid disease, including Graves’ disease [27]. An X chromosome genome-wide association study (GWAS) was conducted by Chu et al. to study the association between the GPR174 gene and Graves’ disease among Han population [27]. In this study, 14,141 single nucleotide polymorphisms on X chromosome were genotyped. Among them, rs3827440 is a non-synonymous single nucleotide polymorphism within the GPR174 gene, with the minor allele frequency being 0.45 in this population, and thus is a functional variant of interest. Further, statistical analysis of both the GWAS data and the replication data showed that rs3827440 is statistically significantly associated with Graves’ disease. At rs3827440, there are two alleles *T* and *C*, where *T* is the susceptible allele which is associated with a higher expression level of the GPR174 gene. Several studies [7, 15] showed that XCI-S is associated with autoimmune thyroid disease. So, we applied the LR, Fieller’s and delta methods to explore if rs3827440 undergoes an XCI-S pattern. We only selected the females to estimate the degree of XCI skewing as well as its 95% CI. In the GWAS stage, 2242 females were sampled (1115 cases and 1127 controls). In the case group, the numbers of females with genotypes *CC*, *TC*, and *TT* are 163, 508, and 444, respectively. Those in the control group are 219, 541, and 367, respectively. In the replication stage, 6260 females were sampled with genotype counts 471, 1606, and 1298 in the case group, and 584, 1344, and 957 in the control group for *CC*, *TC*, and *TT*, respectively. The estimated allele frequency of *T* in females is 0.57 and 0.56 for the GWAS stage and the replication stage, respectively. We applied each of the proposed methods to the data in the GWAS stage and those in the replication stage. After that, we used our proposed methods to deal with the pooled data, by incorporating the stage as a covariate.

Table 5 gives the point estimate $\hat {\gamma }$ and its 95% CIs, based on the LR, Fieller’s and delta methods. From the table, we observe that the LR-type CIs and the Fieller’s CIs are almost the same. The delta-type CIs are nested within the LR-type and Fieller’s CIs for the replication stage and the pooled data, which may be caused by the fact that the delta method underestimates the CP. We also find that the LR-type CI and the Fieller’s CI are asymmetrical around its point estimate in the GWAS stage but are nearly symmetrical around the point estimate in the replication stage and the pooled analysis, which is probably due to the larger sample size in the replication stage and the pooled dataset. In the GWAS stage, the point estimate $\hat {\gamma }$ is 0.957. All of the three types of CIs contain 1 (XCI-R). In the replication stage, the point estimate $\hat {\gamma }$ is 1.513 and all the CIs do not contain 1. The results in the replication stage suggest the XCI-S pattern at rs3827440 with 75.7*%* (1.513/2) of cells having the risk allele *T* active and the other 24.3*%* of cells having the normal allele *C* active. Note that the statistical results for both two stage data suggest different XCI patterns. One possible reason is that the variance of $\hat {\gamma }$ is larger in the GWAS data and there may exist study heterogeneity between those two stages. The results for the pooled data give the point estimate $\hat {\gamma }=1.373$ by adjusting the stage and all of the three types of CIs do not contain 1. This demonstrates that rs3827440 probably undergoes the XCI-S pattern with 68.7*%* (1.373/2) of cells keeping the risk allele *T* active, while the other 31.3*%* of cells keeping the normal allele *C* active. However, this observation needs to be further confirmed by functional analysis of this variant.

**Table 5:** Statistical inference for *γ* at rs3827440 in females based on the LR, Fieller’s and delta methods

		95% CI		
Stage	$\hat {\gamma }$	LR	Fieller	Delta
GWAS	0.957	[0, 1.657]	[0, 1.658]	[0.241, 1.672]
Replication	1.513	[1.123, 1.930]	[1.122, 1.930]	[1.126, 1.900]
Pooled	1.373	[1.028, 1.719]	[1.028, 1.719]	[1.037, 1.708]

## Discussion

In this article, we proposed a statistical measure to estimate the degree of the skewness of XCI (i.e. *γ*). We first showed that *γ* can be expressed as a ratio of two logistic regression coefficients. Then, we constructed a ratio estimate $\hat {\gamma }$ for *γ* and also derived three types of CIs (the LR, Fieller’s and delta methods) to evaluate its variation. The delta method is a simple and non-iterative procedure but generally has poor statistical properties, which is probably caused by the skewness of $\hat {\gamma }$. On the other hand, the LR method and the Fieller’s method are based on a simple reparameterization procedure and thus does not require the normality assumption of $\hat {\gamma }$. The simulation results demonstrate that the LR method and the Fieller’s method have better performance than the delta method. On the other hand, note that the LR-type CI will be close to the Fieller’s CI when *N* is large. In this regard, the Fieller’s CI is preferential since it is a non-iterative procedure. However, when *N* is relatively small, the LR-type CI is recommended for its robustness. In addition, our software SkewXCI is freely available at http://www.echobelt.org/web/UploadFiles/SkewXCI.html, which is implemented in R (http://www.r-project.org/, version 3.5.1).

Our proposed methods have several limitations. First, our methods assume that the genetic effect of the mutant allele among all the cells is additive on the disease. On the other hand, notice that Model (1) under XCI is different from genetic models (dominant, additive, and recessive) on autosomes or on X chromosome under XCI-E. Specifically, genetic model defines the relationship between two alleles at a locus and usually varies from locus to locus [28–30]. However, when XCI occurs, only one allele is active at each locus in each cell and most of the loci share the same XCI pattern. As such, the magnitude of *γ*/2 is a measure of the proportion of cells with the mutant allele active among all the cells in heterozygous females. For instance, adrenoleukodystrophy has been previously viewed as an X-linked recessive disorder where the female carriers are commonly thought to be normal or only mildly affected [31]. However, a recent study showed that the heterozygous females with adrenoleukodystrophy have a wide spectrum of clinical manifestations, ranging from mild to severe phenotypes, which is probably due to the various degree of XCI-S towards the mutant allele. Second, we simply cut the estimated CI within the interval [ 0, 2] and this may lead to potential loss of information. However, if we incorporate this interval constraint into statistical inference, then the LR, Fieller’s and delta methods no longer follow a simple chi-square distribution or a standard normal distribution due to the boundary problem [32]. An alternative method is the Bayesian inference, where such constraint can be regarded as prior information. For instance, when no other information is available, we can choose an uniform prior distribution within the interval [ 0,2] for *γ*. Once the posterior distribution is derived, its percentiles or variance can be used to construct the corresponding CI. Third, note that the validity of our proposed measure is based on the assumption that there exists association between disease and allele *A*. Therefore, in GWAS, we can first screen the associated single nucleotide polymorphisms as candidate loci before making any inference about *γ*. If such association is not statistically significant, our proposed methods may not be reliable. In this situation, according to Fieller’s theorem, the Fieller’s CI and the LR-type CI can be discontinuous as shown in Tables 1, 2, 3, and 4, which is difficult to interpret.

Generally, the LR method and the Fieller’s method have accurate CP and control the ML and MR well, and hence are recommended in practical application. In future work, we will incorporate the information on the interval constraint into analysis so as to further improve the efficiency of the proposed methods. Moreover, we will generalize our methods to quantitative traits.

## Conclusions

When the sample size is greater than 2000, the Fieller’s method has similar performance to the LR method and thus is preferential due to the non-iterative computation procedure. However, the LR method is recommended otherwise because it has better statistical properties, especially in small samples.

## Methods

### Point estimation for ***γ***

Consider an X-linked diallelic locus with normal allele *a* and mutant allele *A*. We only select the females because XCI is unrelated to males. For females, suppose that *aa*, *Aa* and *AA* are three genotypes and let *X*={0, *γ*, 2} be the corresponding genotypic value, respectively, with *γ*∈ [ 0, 2]. For a case-control design, let *Y*=1 (0) denote that the female is affected (unaffected). Then, the association between *Y* and *X* can be expressed using a logistic regression model

1 $$  \text{Logit}(\text{Pr}(Y=1|X,\boldsymbol{z})) =\beta_{0} + \beta X+\boldsymbol{b}^{T}\boldsymbol{z},  $$

where *β*_0_ is the intercept, *β* is the regression coefficient for *X*, ***z*** is a vector of covariates that need to be adjusted (e.g. age), and ***b***^*T*^ is a vector of regression coefficients for ***z***.

To estimate *γ*, we decompose the genotypic value *X* as *X*=*γX*_1_+(2−*γ*)*X*_2_, where *X*_1_=*I*_{*G*=*Aa* or *AA*}_, *X*_2_=*I*_{*G*=*AA*}_, *G* denotes the genotype of the female and *I*_{.}_ is the indicator function. It can be seen that *X*_1_ indicates if the genotype contains the mutant allele *A* and *X*_2_ represents if the genotype is the homozygote *AA*. As such, Model (1) becomes

$$\begin{array}{*{20}l} \text{Logit}(\text{Pr}(Y &= 1|X_{1},X_{2},\boldsymbol{z}))\\ &= \beta_{0} + \beta \gamma X_{1} + \beta (2-\gamma) X_{2}+\boldsymbol{b}^{T}\boldsymbol{z}. \end{array} $$

Let *β*_1_=*βγ* and *β*_2_=*β*(2−*γ*). Then, the above model can be rewritten as

2 $$\begin{array}{*{20}l}  \text{Logit}(\text{Pr}(Y &= 1|X_{1},X_{2},\boldsymbol{z})) \\ &= \beta_{0} + \beta_{1} X_{1} + \beta_{2} X_{2}+\boldsymbol{b}^{T}\boldsymbol{z}. \end{array} $$

Further, due to this reparameterization, *γ* can be represented as

3 $$  \gamma =\frac {2\beta_{1}}{\beta_{1}+\beta_{2}},  $$

when *β*=(*β*_1_+*β*_2_)/2≠0. *γ* can only be well defined in presence of the association between the disease and the allele *A*. Note that *γ*∈ [ 0, 2] means that *β*_1_ and *β*_2_ have the same sign. That is, the genetic effect of heterozygous genotype in females lies between those of two homozygotes, which is generally satisfied in real applications. From Eq. (3), we have *γ*=0 (2) if and only if *β*_1_=0 and *β*_2_≠0 (*β*_1_≠0 and *β*_2_=0), representing XCI-S fully towards the normal (mutant) allele *a* (*A*), while *γ*=1 if and only if *β*_1_=*β*_2_≠0, which means XCI-R. So, if we get the MLEs $\hat {\beta }_{1}$ and $\hat {\beta }_{2}$ of *β*_1_ and *β*_2_, then $\hat {\gamma }=2\hat {\beta }_{1}/(\hat {\beta }_{1}+\hat {\beta }_{2})$ is the MLE of *γ* by the invariance property of MLE.

Note that $\hat {\beta }_{1}$ and $\hat {\beta }_{2}$ can be easily calculated through the standard logistic regression procedure. Specifically, suppose that we collect *N* unrelated females from a homogeneous population. Then, the log-likelihood function of the sample can be written as

$$\begin{array}{*{20}l} &l_{1}\left(\beta_{0},\beta_{1},\beta_{2},\boldsymbol{b}^{T}\right) \\ &=\sum\limits_{i=1}^{N} \left[{y_{i}}\left(\beta_{0}+\beta_{1} x_{i1}+\beta_{2} x_{i2}+\boldsymbol{b}^{T}\boldsymbol{z_{i}}\right)\right.\\ &\quad- \left.\text{log}\left(1+\text{exp}\left(\beta_{0}+\beta_{1} x_{i1}+\beta_{2} x_{i2}+\boldsymbol{b}^{T}\boldsymbol{z}_{i}\right)\right)\right], \end{array} $$

where *y*_*i*_, *x*_*i*1_, *x*_*i*2_ and ***z***_*i*_ respectively are the values of *Y*, *X*_1_, *X*_2_ and ***z*** of female *i*. Then, $\hat {\beta }_{1}$ and $\hat {\beta }_{2}$ are obtained by maximizing the above log-likelihood function, i.e.

$$l_{1}\left(\hat{\beta_{0}},\hat{\beta}_{1},\hat{\beta}_{2},\hat{\boldsymbol{b}}^{T}\right)= {\underset{\beta_{0}, \beta_{1},\beta_{2}, \boldsymbol{b}^{T}}{\arg\max}} \ l_{1}\left(\beta_{0},\beta_{1},\beta_{2},\boldsymbol{b}^{T}\right), $$

where $\hat {\beta _{0}}$ and $\hat {\boldsymbol {b}}^{T}$ are the MLEs of *β*_0_ and ***b***^*T*^, respectively.

### Confidence interval of ***γ*** based on delta method

Once the point estimate of *γ* is derived, we need to calculate the standard error or CI to evaluate its precision. Since $\hat {\gamma }$ is also a ratio estimate, a natural idea is to use the first order Taylor series expansion of $\hat {\gamma }$ and then obtain its asymptotic variance. Specifically, by the consistency of MLE, $\hat {\beta }_{1}$ and $\hat {\beta }_{2}$ are close to *β*_1_ and *β*_2_, respectively, when *N* is large. Note that *β*=(*β*_1_+*β*_2_)/2 and thus *γ* can be rewritten as *β*_1_/*β*. Making a first order Taylor expansion of $\hat {\gamma }$ around the point (*β*_1_, *β*) and evaluating this at $\left (\hat {\beta }_{1},\ \hat {\beta }\right)$, we have

$$\hat{\gamma}\approx\frac {\beta_{1}}{\beta}+\left(\hat{\beta_{1}}-\beta_{1}\right) \frac{1}{\beta}-\left(\hat{\beta}-\beta\right) \frac{\beta_{1}}{\beta^{2}}, $$

where $\hat {\beta }=\left (\hat {\beta _{1}} +\hat {\beta _{2}}\right)/2$. Taking variance from both sides, the above equation becomes

4 $$\begin{array}{*{20}l}  \text{Var}(\hat{\gamma})&\approx \frac{1}{\beta^{2}}\text{Var}\left(\hat{\beta_{1}}\right)\\ &+\frac{\beta_{1}^{2}}{\beta^{4}}\text{Var}\left(\hat{\beta}\right)- \frac{2\beta_{1}}{\beta^{3}}\text{Cov}\left(\hat{\beta_{1}},\ \hat{\beta}\right). \end{array} $$

Notice that

$$\text{Var}\left(\hat{\beta}\right)=\frac{1}{4}\left(\text{Var}\left(\hat{\beta_{1}}\right)+\text{Var}\left(\hat{\beta_{2}}\right)+2\mbox{Cov}\left(\hat{\beta_{1}},\ \hat{\beta_{2}}\right)\right) $$

and

$$\text{Cov}\left(\hat{\beta_{1}},\ \hat{\beta}\right)=\frac{1}{2}\left({\text{Var}}\left(\hat{\beta_{1}}\right)+ \text{Cov}\left(\hat{\beta_{1}},\ \hat{\beta_{2}}\right)\right), $$

where $\text {Var}\left (\hat {\beta _{1}}\right)$, $\text {Var}\left (\hat {\beta _{2}}\right)$ and ${\text {Cov}}\left (\hat {\beta _{1}},\hat {\beta _{2}}\right)$ are the elements of the variance-covariance matrix ***V*** of $\hat {\beta _{1}}$ and $\hat {\beta _{2}}$. Generally, ***V*** has no simple form when covariates are included in the model, but can be derived from the empirical Fisher’s information matrix $\boldsymbol {\hat {I}}$ for (*β*_0_, *β*_1_, *β*_2_, ***b***^*T*^)^*T*^ [33]. For Model (2),

$$\boldsymbol{\hat{I}}=\boldsymbol{U^{T}\hat{W}U}, $$

where ***U***=(***1***, ***X***_***1***_, ***X***_***2***_, ***z***) is the design matrix, ***X***_***1***_=(*x*_11_,*x*_21_,...,*x*_*N*1_)^*T*^, ***X***_***2***_=(*x*_12_,*x*_22_,...,*x*_*N*2_)^*T*^, ***z***=(***z***_1_,***z***_2_,...,***z***_*N*_)^*T*^, $\boldsymbol {\hat {W}}={\text {diag}}\left (\hat {w}_{1},\hat {w}_{2},...,\hat {w}_{N}\right)$ is a diagonal matrix with diagonal elements

$$\hat{w}_{i}=\hat{f}_{i} \left(1-\hat{f}_{i}\right)\ (i=1,2,...,N), $$

and

$$\hat{f}_{i}=\frac{\text{exp}\left(\hat{\beta}_{0} + \hat{\beta}_{1} x_{i1} + \hat{\beta}_{2} x_{i2} +\boldsymbol{\hat{b}}^{T}\boldsymbol{z}_{i}\right)}{1+{\text{exp}}\left(\hat{\beta}_{0} + \hat{\beta}_{1} x_{i1} + \hat{\beta}_{2} x_{i2} +\boldsymbol{\hat{b}}^{T}\boldsymbol{z}_{i}\right)} $$

represents the estimated penetrances for female *i*. Once $\boldsymbol {\hat {I}}$ is estimated, the partial information matrix $\boldsymbol {\hat {I}}_{1}$ for *β*_1_ and *β*_2_ given *β*_0_ and ***b***^*T*^ can be computed and thus $\boldsymbol {V}=\boldsymbol {\hat {I}}^{-1}_{1}$. If there is no covariate in the model, then ***V*** has the following form [see Appendix A of Additional file 1]

$$\left(\begin{array}{cc} \frac{1}{n_{aa} \hat{w}_{aa}}+\frac{1}{n_{Aa} \hat{w}_{Aa}} & -\frac{1}{n_{Aa} \hat{w}_{Aa}}\\ -\frac{1}{n_{Aa} \hat{w}_{Aa}} & \frac{1}{n_{Aa} \hat{w}_{Aa}}+ \frac{1}{n_{AA} \hat{w}_{AA}} \end{array} \right), $$

where *n*_*aa*_, *n*_*Aa*_ and *n*_*AA*_ are the numbers of the females with *aa*, *Aa* and *AA*, respectively, and *N*=*n*_*aa*_+*n*_*Aa*_+*n*_*AA*_; $\hat {w}_{aa}$, $\hat {w}_{aa}$ and $\hat {w}_{aa}$ are the weighted elements for *aa*, *Aa* and *AA*, respectively, with

$$\hat{w}_{G}=\hat{f}_{G} \left(1-\hat{f}_{G}\right) \ (G=aa,Aa, \text{or} AA), $$

and

$$\begin{array}{*{20}l} \hat{f}_{aa} &=\frac{\text{exp}\left(\hat{\beta}_{0}\right)}{1+\text{exp}\left(\hat{\beta}_{0}\right)},\\ \hat{f}_{Aa} &=\frac{\text{exp}\left(\hat{\beta}_{0} + \hat{\beta}_{1}\right)}{1+\text{exp}\left(\hat{\beta}_{0}+\hat{\beta}_{1}\right)} \end{array} $$

and

$$\hat{f}_{AA} =\frac{\text{exp}\left(\hat{\beta}_{0}+ \hat{\beta}_{1}+\hat{\beta}_{2}\right)}{1+\text{exp}\left(\hat{\beta}_{0}+\hat{\beta}_{1}+\hat{\beta}_{2}\right)} $$

representing the estimated penetrances for *aa*, *Aa* and *AA*, respectively.

Replacing *β* and *β*_1_ by $\hat {\beta }$ and $\hat {\beta _{1}}$ in Eq. (4), we estimate the delta-type standard error [34]

$$\begin{array}{*{20}l} \hat{\text{Var}}\left(\hat{\gamma}\right)&\approx\frac{1}{\hat{\beta}^{2}}\text{Var}\left(\hat{\beta_{1}}\right)\\ &+\frac{\hat{\beta}_{1}^{2}}{\hat{\beta}^{4}}\text{Var}\left(\hat{\beta}\right)- \frac{2\hat{\beta}_{1}}{\hat{\beta}^{3}}\text{Cov}\left(\hat{\beta_{1}},\ \hat{\beta}\right). \end{array} $$

As such, the delta-type CI $\left (\gamma _{L}^{d},\gamma _{U}^{d}\right)$ at level (1−*α*) can be expressed as

$$\begin{array}{*{20}l} \left(\ \hat{\gamma}-Z_{1-\frac{\alpha}{2}} \sqrt{\hat{\text{Var}}\left(\hat{\gamma}\right)},\ \hat{\gamma}+Z_{1-\frac{\alpha}{2}} \sqrt{\hat{\text{Var}}\left(\hat{\gamma}\right)}\ \right), \end{array} $$

where *Z*_1−*α*/2_ denotes the (1−*α*/2)-quantile of the standard normal distribution. Note that the estimated CI may be out of the range of [0, 2] when the variation is large, which should be cut off. To test the null hypothesis *H*_0_:*γ*=*γ*_0_ against the alternative hypothesis *H*_1_:*γ*≠*γ*_0_, we have

$$\frac{\hat{\gamma}-\gamma_{0}}{\sqrt{\text{Var}\left(\hat{\gamma}\right)}} \sim N(0,1) $$

under *H*_0_, where *γ*_0_ is an arbitrary constant between [ 0, 2], such as 1 (XCI-R).

The delta method is a non-iterative procedure and thus is easy to be implemented. However, the CI of a ratio estimate is generally skewed, while the delta-type CI is symmetrical [35, 36]. Therefore, it is necessary to propose the Fieller’s and likelihood ratio methods to overcome this shortcoming in the following sections.

### Confidence interval of ***γ*** based on Fieller’s method

The Fieller’s method is another widely used non-iterative approach for constructing CI for ratio estimate [37]. This type of CI can be asymmetrical around $\hat {\gamma }$. To propose the Fieller’s CI, we first need to build a Wald test for testing *γ*=*γ*_0_. Specifically, under *γ*=*γ*_0_, we have *β*_1_−*γ*_0_*β*=0. Therefore, the Wald test for testing *γ*=*γ*_0_ can be written as

$$\frac{\hat{\beta}_{1}-\gamma_{0} \hat{\beta}}{\sqrt{\text{Var}\left(\hat{\beta}_{1}\right)+\gamma_{0}^{2} \text{Var}\left(\hat{\beta}\right) -2\gamma_{0} \text{Cov}\left(\hat{\beta_{1}},\ \hat{\beta}\right) }}, $$

which follows a standard normal distribution. Then, the confidence limits $\gamma _{L}^{f}$ and $\gamma _{U}^{f} \left (\gamma _{L}^{f}<\gamma _{U}^{f}\right)$ for Fieller’s CI at level (1−*α*) can be found by solving the following equation

$$\frac{\hat{\beta}_{1}-\gamma_{0} \hat{\beta}}{\sqrt{\text{Var}\left(\hat{\beta}_{1}\right)+\gamma_{0}^{2} \text{Var}\left(\hat{\beta}\right) -2\gamma_{0} \text{Cov}\left(\hat{\beta_{1}},\ \hat{\beta}\right) }}=Z_{1-\frac{\alpha}{2}}. $$

Rearranging the above equation yields a quadratic equation with respect to *γ*_0_

$$D\gamma_{0}^{2}+E \gamma_{0}+F=0, $$

where

$$\begin{array}{*{20}l} D&=\hat{\beta}^{2}-Z_{1-\frac{\alpha}{2}}^{2} \text{Var}\left(\hat{\beta}\right), \\ E&=2 \left(Z_{1-\frac{\alpha}{2}}^{2}\text{Cov}\left(\hat{\beta_{1}},\ \hat{\beta}\right) - \hat{\beta}_{1} \hat{\beta} \right) \end{array} $$

and

$$F=\hat{\beta}_{1}^{2}-Z_{1-\frac{\alpha}{2}}^{2} \text{Var}\left(\hat{\beta}_{1}\right). $$

Suppose *Δ*=*E*^2^−4*DF*>0, then this equation must have two unequal roots with $\gamma _{L}^{f}$ and $\gamma _{U}^{f}$ being $\left (-E\pm \sqrt {\Delta }\right)/2D$. According to Fieller’s theorem, we know that *D*>0 implies *Δ*>0. In this situation, the Fieller’s CI is continuous and can be denoted by $\left (\gamma _{L}^{f}, \gamma _{U}^{f}\right)$. Note that *D*>0 is equivalent to $\left |\hat {\beta }/\sqrt {\text {Var}\left (\hat {\beta }\right)}\right | >Z_{1-\frac {\alpha }{2}}$. That is, there exists statistically significant association between the disease and the allele *A* at the significance level *α*. However, if there is no such association (i.e. *D*<0), the Fieller’s CI will be unbounded. For instance, if *Δ*<0, the Fieller’s CI will be (−*∞*,*∞*). If *Δ*>0, the Fieller’s CI will be $\left (-\infty, \gamma _{L}^{f}\right)\bigcup \left (\gamma _{U}^{f},\infty \right)$, which is the discontinuous CI. In real applications, it generally makes little sense to infer about *γ* if there is no association between the disease and the allele *A* according to its definition. In addition, the Fieller’s CI should also be restricted to the interval [0,2] when needed.

The Fieller’s method usually demonstrates better coverage probability than the delta method. Notice that the Fieller’s CI is based on the inversion of the Wald test. Since the LR test is expected to have more robust properties in small samples, so it is desirable to propose the LR method in the next section.

### Confidence interval of ***γ*** based on likelihood ratio method

To obtain the LR-based CI, we first construct a likelihood ratio test for testing *γ*=*γ*_0_. As mentioned above, we have derived the MLEs $\hat {\beta }_{0}$, $\hat {\beta }_{1}$, $\hat {\beta }_{2}$ and $\hat {\boldsymbol {b}}^{T}$ of *β*_0_, *β*_1_, *β*_2_ and ***b***^*T*^ under *H*_1_. To calculate the likelihood ratio test statistic *λ*, we further evaluate the likelihood function under *H*_0_:*γ*=*γ*_0_. If *H*_0_ holds, the genotypic value *X* equals 0, *γ*_0_ and 2 for *aa*, *Aa* and *AA*, respectively. In this regard, Model (1) is reduced to be a standard logistic model and the log-likelihood function under *H*_0_ can be written as

$$\begin{array}{*{20}l} l_{0}\left(\beta_{0},\beta,\boldsymbol{b}^{T}\right)&= \sum\limits_{i=1}^{N} \left[{y_{i}}\left(\beta_{0}+\beta x_{i}+\boldsymbol{b}^{T}\boldsymbol{z_{i}}\right)\right.\\ &\quad-\left. \text{log}\left(1+\text{exp}\left(\beta_{0}+\beta x_{i}+\boldsymbol{b}^{T}\boldsymbol{ z}_{i}\right)\right)\right]\!, \end{array} $$

where *x*_*i*_ is the genotypic value of *X* of female *i*. Let $\tilde {\beta }_{0}$, $\tilde {\beta }$ and $\tilde {\boldsymbol {b}}^{T}$ be the MLEs of *β*_0_, *β* and ***b***^*T*^ under *H*_0_, respectively. Then,

$$l_{0}\left(\tilde{\beta}_{0},\tilde{\beta},\tilde{\boldsymbol{b}}^{T}\right)= {\underset{\beta_{0}, \beta, \boldsymbol{b}^{T}}{\arg\max}} \ l_{0}\left(\beta_{0},\beta,\boldsymbol{b}^{T}\right), $$

and *λ* can be computed as

$$\lambda=2\left(l_{1}\left(\hat{\beta_{0}},\hat{\beta}_{1},\hat{\beta}_{2},\hat{\boldsymbol{b}}^{T}\right) -l_{0}\left(\tilde{\beta}_{0},\tilde{\beta},\tilde{\boldsymbol{b}}^{T}\right)\right). $$

*λ* asymptotically follows a chi-square distribution with the degree of freedom being one $\left (\text {i.e.}\, \chi _{1}^{2}\right)$.

Now, we introduce how to obtain the LR-type CI. For each given *γ*_0_, we can calculate the corresponding value of the log-likelihood function *l*_0_ and $\tilde {\boldsymbol {\theta }}=\left (\tilde {\beta }_{0}, \tilde {\beta }, \tilde {\boldsymbol {b}}^{T}\right)^{T}$ under *H*_0_. So, *l*_0_ and $\tilde {\boldsymbol {\theta }}$ are single variable functions with respect to *γ*_0_ and can be denoted by *l*_0_=*l*_0_(*γ*_0_) and $\tilde {\boldsymbol {\theta }}=\tilde {\boldsymbol {\theta }}(\gamma _{0})$, respectively. At the significance level *α*, the confidence limits $\gamma _{L}^{l}$ and $\gamma _{U}^{l} \left (\gamma _{L}^{l}<\gamma _{U}^{l}\right)$ of the LR-type CI is determined by the following equation with respect to *γ*_0_

5 $$  l_{0}(\gamma_{0})-l_{1}\left(\hat{\beta_{0}},\hat{\beta}_{1},\hat{\beta}_{2},\hat{\boldsymbol{b}}^{T}\right)+\frac{q_{1-\alpha}}{2}=0,  $$

where *q*_1−*α*_ denotes the (1−*α*)-quantile of the $\chi _{1}^{2}$ distribution. Obviously, Eq. (5) has no closed form solutions and numerical method can be adopted. Note that ***θ***=(*β*_0_,*β*,***b***^*T*^)^*T*^ are nuisance parameters in Eq. (5) which depend on *γ*_0_. To solve this equation, it generally requires several iterations with different values of *γ*_0_, and for each *γ*_0_, the iterative maximization over the remaining parameters is also needed to determine $\tilde {\boldsymbol {\theta }}$. This procedure is relatively time-consuming. Therefore, to reduce the computational burden, borrowing the idea of Venzon and Moolgavkar [38], we can find the roots of Eq. (5) by solving the following system of non-linear equations

$$\begin{array}{*{20}l} l_{0}(\gamma_{0}, \boldsymbol{\theta})-l_{1}\left(\hat{\beta_{0}},\hat{\beta}_{1},\hat{\beta}_{2},\hat{\boldsymbol{b}}^{T}\right)+\frac{q_{1-\alpha}}{2}&=0 \\ \frac{\partial l_{0}}{\partial \boldsymbol{\theta}}(\gamma_{0}, \boldsymbol{\theta})&=\boldsymbol{0}, \end{array} $$

which is easily implemented in the commonly used software (e.g. nleqslv package in R). Note that the above system differs only in the first equation from the system (with the first equation being replaced by $\frac {\partial l_{0}}{\partial \gamma _{0}}(\gamma _{0}, \boldsymbol {\theta })=0$) that defines the MLEs $\hat {\gamma }$ and $\hat {\boldsymbol {\theta }}=\left (\hat {\beta }_{0}, \hat {\beta }, \hat {\boldsymbol {b}}^{T}\right)^{T}$. Therefore, finding a root of such system almost has the same difficulty as that of finding the MLEs of Model (2) [38]. As such, this algorithm is generally more efficient.

On the other hand, based on the fact that the Wald test and the LR test are asymptotically equivalent in large samples, we know that the confidence limits of the Fieller’s CI and the LR-type CI should be close to each other. Therefore, we used the confidence limits of the Fieller’s CI as the initial values for *γ*_0_. For example, when searching the lower limit, we chose the initial values for *γ*_0_ and ***θ*** as $\gamma _{L}^{f}$ and $\tilde {\boldsymbol {\theta }}\!\left (\gamma _{L}^{f}\right)$, respectively, where $\tilde {\boldsymbol {\theta }}\!\left (\gamma _{L}^{f}\right)$ can be computed from the standard logistic regression procedure. Similarly, we used the same strategy to search the upper limit. The algorithm based on this choice of the initial values works well in most situations. However, in some scenarios, the Fieller’s CI and the LR-type CI may be very different. Thus, using the confidence limits of the Fieller’s CI as the initial values may cause that the algorithm does not converge. In this regard, we should directly solve the single variable function of Eq. (5). For example, we can use the bisection method to find the roots of Eq. (5) within the interval [ 0,2] (e.g. rootSolve package in R).

Like the Fieller’s CI, the LR-type CI can be unbounded when there is no association between the disease and the allele *A*. Specifically, when Equation (5) has no root, then the LR-type CI will be (−*∞*,*∞*). Otherwise, there will be two roots $\gamma _{L}^{l}$ and $\gamma _{U}^{l}$. If $\gamma _{L}^{l}<\hat {\gamma }<\gamma _{U}^{l}$, then the LR-type CI is continuous and can be represented as $\left (\gamma _{L}^{l},\gamma _{U}^{l}\right)$. If $\hat {\gamma }\not \in \left (\gamma _{L}^{l},\gamma _{U}^{l}\right)$, then the LR-type CI will be $\left (-\infty, \gamma _{L}^{l}\right)\bigcup \left (\gamma _{U}^{l},\infty \right)$, which is the discontinuous CI. Similar to the delta and Fieller’s methods, the LR-type CI is also truncated by [ 0, 2] if necessary.

The LR-based CI and the Fieller’s CI can be asymmetrical which is an appealing choice, compared to the delta method. This is because the distribution of a ratio estimate is generally non-normal with a heavy tail, especially when *N* is small. Additionally, it will be quite straightforward to incorporate covariates using the LR method.

### Simulation settings

For simplicity, we assumed that there is no covariate included in the model in our simulation study. We incorporated the covariate into the real data analysis later. For a case-control design, we presumed that the genotype distribution in the case group and that in the control group of females follow trinomial distributions with probabilities (*h*_0_, *h*_1_, *h*_2_) and (*g*_0_, *g*_1_, *g*_2_), respectively, where *h*_0_ (*g*_0_), *h*_1_ (*g*_1_) and *h*_2_ (*g*_2_) are the frequencies of *aa*, *Aa* and *AA* in the case (control) group, respectively. Let *h*_0_/*g*_0_=*m*, *h*_1_/*g*_1_=*λ*_1_(*h*_0_/*g*_0_)=*λ*_1_*m* and *h*_2_/*g*_2_=*λ*_2_(*h*_0_/*g*_0_)=*λ*_2_*m*, where *λ*_1_ and *λ*_2_ are the odds ratios for *Aa* and *AA* compared to *aa* in females, respectively. By *h*_0_+*h*_1_+*h*_2_=1, we have *m*=1/(*g*_0_+*λ*_1_*g*_1_+*λ*_2_*g*_2_), *h*_0_=*g*_0_/(*g*_0_+*λ*_1_*g*_1_+*λ*_2_*g*_2_), *h*_1_=*λ*_1_*g*_1_/(*g*_0_+*λ*_1_*g*_1_+*λ*_2_*g*_2_) and *h*_2_=*λ*_2_*g*_2_/(*g*_0_+*λ*_1_*g*_1_+*λ*_2_*g*_2_). Thus, (*h*_0_, *h*_1_, *h*_2_) is calculated by (*g*_0_, *g*_1_, *g*_2_), *λ*_1_ and *λ*_2_. The value of (*g*_0_, *g*_1_, *g*_2_) is determined by the allele frequency of *A* (denoted by *p*=1−*q*) and the inbreeding coefficient *ρ*, i.e. *g*_0_=*q*^2^+*ρpq*, *g*_1_=2(1−*ρ*)*pq* and *g*_2_=*p*^2^+*ρpq*. Specifically, *ρ*=0 implies that Hardy-Weinberg equilibrium holds in the control group and *ρ*≠0 indicates Hardy-Weinberg disequilibrium. Furthermore, from Model (2), *β*_0_=log(*m*), *β*_1_=log(*λ*_1_) and *β*_1_+*β*_2_=log(*λ*_2_). Then, *γ*=2log(*λ*_1_)/log(*λ*_2_), i.e. $\lambda _{1}=\lambda _{2}^{\gamma /2}$. As such, we defined the simulation settings as follows: *p* is fixed at 0.1 and 0.3, and *ρ* is set to be 0 and 0.05. The true value of *γ* is fixed at 0, 0.5, 1, 1.5 and 2. *λ*_2_ is assigned to be 1.5 and 2. We selected *N* as 500 (2000) where both the case and control groups have 250 (1000) females. The confidence level is fixed at (1−*α*)=95*%* and the number of replications *k* is 10,000.

We compared the performance of three types of CIs based on CP, ML, MR, ML/(ML+MR) and DP. CP is defined to be the proportion that the CI contains the true value of *γ* among *k* replications, regardless of whether the CI is continuous or not. ML and MR are calculated by $\text {ML}=\#\left [(\gamma _{0}<\gamma _{L})\cap \left (\gamma _{L}\le \hat {\gamma }\le \gamma _{U}\right)\right ]/k$, and $\text {MR}=\#\left [(\gamma _{0}>\gamma _{U})\cap \left (\gamma _{L}\le \hat {\gamma }\le \gamma _{U}\right)\right ]/k$, respectively, where *#* denotes the counting measure, and *γ*_*L*_ and *γ*_*U*_ are the confidence limits of the estimated CI. Note that $\gamma _{L}\le \hat {\gamma }\le \gamma _{U}$ means that the CI is continuous. As such, we only consider the continuous CIs when estimating ML and MR, since it is impossible to distinguish between the left side and the right side if the CI is discontinuous. Further, DP is computed as $1-\#(\gamma _{L}\le \hat {\gamma }\le \gamma _{U})/k$. We believed that a good CI should control the CP well as well as have the balanced ML and MR. ML and MR are used together to measure the location of CI. If a balance between ML and MR is achieved, then ML/(ML+MR) is close to 0.5. On the other hand, note that the delta-type CI is always bounded. Therefore, the DP for the delta method will stay at 0. However, for the Fieller’s CI and the LR-type CI, small DP is desirable.

## Additional file

Additional file 1 Appendices and Supplementary tables and figures. **Appendix A** Derivation of the closed form of ***V*** without covariates; **Appendix B** Size and power results for testing *γ*=*γ*_0_; **Tables S1-S2** Estimated CP (*%*), ML (*%*), MR (*%*), ML/(ML+MR) and DP (*%*) of the two-sided 95*%* CI when *N*=500, *ρ*=0.05, and *λ*_2_=1.5 or 2 for the LR, Fieller’s and delta methods, respectively; **Tables S3-S4** Estimated CP (*%*), ML (*%*), MR (*%*), ML/(ML+MR) and DP (*%*) of the two-sided 95*%* CI when *N*=2000, *ρ*=0.05, and *λ*_2_=1.5 or 2 for the LR, Fieller’s and delta methods, respectively; **Tables S5-S8** Estimated size (*%*) for testing *H*_0_: *γ*=*γ*_0_ with *N*=500 or 2000, and *ρ*=0 or 0.05 for the LR, Fieller’s and delta methods, respectively; **Figures S1-S6** Power comparison of the LR, Fieller’s and delta methods against *γ*. The simulation is based on 10,000 replicates with *N*=500, *ρ*=0 or 0.05, and *γ*_0_=0, 1 or 2, respectively; **Figures S7-S12** Power comparison of the LR, Fieller’s and delta methods against *γ*. The simulation is based on 10,000 replicates with *N*=2000, *ρ*=0 or 0.05, and *γ*_0_=0, 1 or 2, respectively. (PDF 117 kb)

## Abbreviations

CI: Confidence interval
CP: Coverage probability
DP: Proportion of discontinuous CIs
GWAS: Genome-wide association study
LR: Likelihood ratio
ML: Left tail error
MLE: Maximum likelihood estimate
MR: Right tail error
XCI; X chromosome inactivation; XCI-E: Escape from X chromosome inactivation
XCI-R; Random X chromosome inactivation; XCI-S: Skewed X chromosome inactivation
