# Supplementary material for: A statistical measure for the skewness of X chromosome inactivation based on case-control design
Source: BMC Bioinformatics. 2019 Jan 7;20:11. doi: 10.1186/s12859-018-2587-2 (PMC6323862; doi:10.1186/s12859-018-2587-2)
Supplement: Supplementary file 1 — Appendices and Supplementary tables and figures. Appendix A Derivation of the closed form of V without covariates; Appendix B Size and power results for testing γ=γ0; Tables S1-S2 Estimated CP (%), ML (%), MR (%), ML/(ML+MR) and DP (%) of the two-sided 95% CI when N=500, ρ=0.05, and λ2=1.5 or 2 for the LR, Fieller’s and delta methods, respectively; Tables S3-S4 Estimated CP (%), ML (%), MR (%), ML/(ML+MR) and DP (%) of the two-sided 95% CI when N=2000, ρ=0.05, and λ2=1.5 or 2 for the LR, Fieller’s and delta methods, respectively; Tables S5-S8 Estimated size (%) for testing H0: γ=γ0 with N=500 or 2000, and ρ=0 or 0.05 for the LR, Fieller’s and delta methods, respectively; Figures S1-S6 Power comparison of the LR, Fieller’s and delta methods against γ. The simulation is based on 10,000 replicates with N=500, ρ=0 or 0.05, and γ0=0, 1 or 2, respectively; Figures S7-S12 Power comparison of the LR, Fieller’s and delta methods against γ. The simulation is based on 10,000 replicates with N=2000, ρ=0 or 0.05, and γ0=0, 1 or 2, respectively. (PDF 117 kb) [file 12859_2018_2587_MOESM1_ESM.pdf]

Additional file 1

Appendices

Appendix A: Derivation of the closed form of $\boldsymbol{V}$ without covariates

Without covariates, we have $\boldsymbol{U} = (\mathbf{1},\ \boldsymbol{X_1},\ \boldsymbol{X_2})$ with $\boldsymbol{X_1} = (x_{11}, x_{21}, ..., x_{N1})^T$ and $\boldsymbol{X_2} = (x_{12}, x_{22}, ..., x_{N2})^T$. Then, the empirical Fisher's information matrix $\hat{\boldsymbol{I}}$ for $(\beta_0,\ \beta_1,\ \beta_2)^T$ has the form

$$\hat{\boldsymbol{I}} = \boldsymbol{U^T}\hat{\boldsymbol{W}}\boldsymbol{U} = \begin{pmatrix} \sum_{i=1}^{N} \hat{w}_i & \sum_{i=1}^{N} \hat{w}_i x_{i1} & \sum_{i=1}^{N} \hat{w}_i x_{i2} \\ \sum_{i=1}^{N} \hat{w}_i x_{i1} & \sum_{i=1}^{N} \hat{w}_i x_{i1}^2 & \sum_{i=1}^{N} \hat{w}_i x_{i1} x_{i2} \\ \sum_{i=1}^{N} \hat{w}_i x_{i2} & \sum_{i=1}^{N} \hat{w}_i x_{i1} x_{i2} & \sum_{i=1}^{N} \hat{w}_i x_{i2}^2 \end{pmatrix}.$$

Note that

$$\begin{aligned} &\sum_{i=1}^{N} \hat{w}_i = n_{aa}\hat{w}_{aa} + n_{Aa}\hat{w}_{Aa} + n_{AA}\hat{w}_{AA}, \\ &\sum_{i=1}^{N} \hat{w}_i x_{i1} = \sum_{i=1}^{N} \hat{w}_i x_{i1}^2 = n_{Aa}\hat{w}_{Aa} + n_{AA}\hat{w}_{AA}, \\ &\sum_{i=1}^{N} \hat{w}_i x_{i2} = \sum_{i=1}^{N} \hat{w}_i x_{i2}^2 = \sum_{i=1}^{N} \hat{w}_i x_{i1} x_{i2} = n_{AA}\hat{w}_{AA}. \end{aligned}$$

Therefore, $\hat{\boldsymbol{I}}$ is reduced to be

$$\hat{\boldsymbol{I}} = \begin{pmatrix} n_{aa}\hat{w}_{aa} + n_{Aa}\hat{w}_{Aa} + n_{AA}\hat{w}_{AA} & n_{Aa}\hat{w}_{Aa} + n_{AA}\hat{w}_{AA} & n_{AA}\hat{w}_{AA} \\ n_{Aa}\hat{w}_{Aa} + n_{AA}\hat{w}_{AA} & n_{Aa}\hat{w}_{Aa} + n_{AA}\hat{w}_{AA} & n_{AA}\hat{w}_{AA} \\ n_{AA}\hat{w}_{AA} & n_{AA}\hat{w}_{AA} & n_{AA}\hat{w}_{AA} \end{pmatrix}.$$

The partial information matrix $\hat{\boldsymbol{I}}_{\mathbf{1}}$ for β_1 and β_2 given β_0 is

$$\hat{\boldsymbol{I}}_{\mathbf{1}} = \begin{pmatrix} n_{Aa}\hat{w}_{Aa} + n_{AA}\hat{w}_{AA} & n_{AA}\hat{w}_{AA} \\ n_{AA}\hat{w}_{AA} & n_{AA}\hat{w}_{AA} \end{pmatrix}$$

$$- \begin{pmatrix} n_{Aa}\hat{w}_{Aa} + n_{AA}\hat{w}_{AA} \\ n_{AA}\hat{w}_{AA} \end{pmatrix} (n_{aa}\hat{w}_{aa} + n_{Aa}\hat{w}_{Aa} + n_{AA}\hat{w}_{AA})^{-1} \begin{pmatrix} n_{Aa}\hat{w}_{Aa} + n_{AA}\hat{w}_{AA} & n_{AA}\hat{w}_{AA} \end{pmatrix}$$

$$= \frac{1}{n_{aa}\hat{w}_{aa} + n_{Aa}\hat{w}_{Aa} + n_{AA}\hat{w}_{AA}} \begin{pmatrix} n_{aa}\hat{w}_{aa}(n_{Aa}\hat{w}_{Aa} + n_{AA}\hat{w}_{AA}) & n_{aa}\hat{w}_{aa}n_{AA}\hat{w}_{AA} \\ n_{aa}\hat{w}_{aa}n_{AA}\hat{w}_{AA} & n_{AA}\hat{w}_{AA}(n_{aa}\hat{w}_{aa} + n_{Aa}\hat{w}_{Aa}) \end{pmatrix}.$$

Then, $\boldsymbol{V}$ is the inversion of $\hat{\boldsymbol{I}}_{\mathbf{1}}$, i.e.

$$\boldsymbol{V} = \begin{pmatrix} \frac{1}{n_{aa}\hat{w}_{aa}} + \frac{1}{n_{Aa}\hat{w}_{Aa}} & -\frac{1}{n_{Aa}\hat{w}_{Aa}} \\ -\frac{1}{n_{Aa}\hat{w}_{Aa}} & \frac{1}{n_{Aa}\hat{w}_{Aa}} + \frac{1}{n_{AA}\hat{w}_{AA}} \end{pmatrix}.$$

Appendix B: Size and power results for testing $\gamma = \gamma_0$

The settings for simulating size and power for testing $\gamma = \gamma_0$ are the same as those in the main text. Note that γ_0 is taken to be 0, 0.5, 1, 1.5 and 2 for simulating the empirical size with $\gamma = \gamma_0$ but is set to be 0, 1 and 2 for estimating the power with $\gamma \neq \gamma_0$. Due to the equivalence between CI and the corresponding hypothesis testing in statistical inference, we know that the size equals (1 − CP). Tables S5 and S6 show the estimated size for testing $H_0: \gamma = \gamma_0$ with $\rho = 0$, $p = 0.1$ and 0.3, and $\lambda_2 = 1.5$ and 2 for the LR, Fieller's and delta methods when $N = 500$ and 2000, respectively. It can be seen from the tables that the LR method and the Fieller's method control the size well except for $N = 500$ and $p = 0.1$, regardless of $\lambda_2 = 1.5$ or 2. Under the scenario of $N = 500$ and $p = 0.1$, both the LR and Fieller's methods are conservative, but the size of the LR method is closer to the significance level. Besides, the delta method has the worst performance in size as the values of its size are either conservative or inflated under all the situations. All the other size results with $\rho = 0.05$ are given in Tables S7 and S8, which are similar to Tables S5 and S6 except for the situation with $N = 500$ and $p = 0.1$, indicating that Hardy-Weinberg disequilibrium has limited effect on the results. Like the CP results, the values of size with ρ being 0.05 are more accurate than those with ρ being 0 under the scenario of $N = 500$ and $p = 0.1$.

Figs. S1-S3 plot the estimated powers with $N = 500$, $\rho = 0$, and γ_0 being 0, 1 and 2 for the LR, Fieller's and delta methods, respectively. From these figures, we find that when p changes from 0.1 to 0.3 or λ_2 changes from 1.5 to 2, both the LR method and the Fieller's method become more powerful, but the delta method does not have this property which is probably due to its inflated size. The powers of the LR method are generally slightly higher than those of the Fieller's method when $p = 0.1$, while they are close to each other when $p = 0.3$. Fig. S1 shows that the delta method performs better in power than other two methods when $p = 0.1$. For $p = 0.3$, the delta method is less powerful than the others when

$\gamma = 0.5$ and 1, but is more powerful when $\gamma = 1.5$ and 2. We can see similar patterns in Fig. S2 to Fig. S1(b) and (d). It is shown in Fig. S3 that the LR and Fieller's methods are more powerful than the delta method when $p = 0.3$. But when $p = 0.1$, the delta method has higher power than the others when $\gamma = 1$ and 1.5, and $\lambda_2 = 1.5$, or $\gamma = 1.5$ and $\lambda_2 = 2$. Figs. S4-S6 display the estimated powers with $N = 2000$, $\rho = 0$, and γ_0 being 0, 1 and 2 for the LR, Fieller's and delta methods, respectively. Those figures are analogous to Figs. S1-S3. It can be seen that when the sample size increases from 500 to 2000, all the powers are larger. In the situation with $N = 2000$, the power profiles of the LR method and the Fieller's method appear to be consistent with each other. Note that the delta method cannot control the size well. Thus, its corresponding estimated power may not be so reliable. All the other power results with $\rho = 0.05$ are given in Figs. S7-S12. The results for the nonzero ρ value are similar to those when $\rho = 0$, and Hardy-Weinberg disequilibrium has little effect on the power results.

Table S1 Estimated CP (%), ML (%), MR (%), ML/(ML+MR) and DP (%) of the two-sided 95% CI when $N = 500$, $\rho = 0.05$, and $\lambda_2 = 1.5$ for the LR, Fieller's and delta methods

		LR				Fieller				Delta			
p	γ	CP	(ML, MR)	$\frac{ML}{ML+MR}$	DP	CP	(ML, MR)	$\frac{ML}{ML+MR}$	DP	CP	(ML, MR)	$\frac{ML}{ML+MR}$	DP
0.1	0	94.93	(4.37, 0.70)	0.86	1.57	95.12	(4.38, 0.50)	0.90	2.18	100	(0, 0)	—	0
	0.5	94.63	(1.96, 2.49)	0.44	1.39	95.37	(2.04, 1.37)	0.60	1.64	95.73	(0, 4.27)	0	0
	1	94.92	(1.06, 3.39)	0.24	1.49	97.04	(1.06, 1.26)	0.46	1.64	82.62	(0, 17.38)	0	0
	1.5	95.19	(0.58, 4.05)	0.13	1.58	97.66	(0.52, 1.62)	0.24	1.81	75.23	(0, 24.77)	0	0
	2	94.85	(0, 5.15)	0	1.79	97.69	(0, 2.31)	0	2.06	70.90	(0, 29.10)	0	0
0.3	0	94.99	(3.62, 1.39)	0.72	1.48	95.05	(3.59, 1.36)	0.73	1.49	99.13	(0.87, 0)	1	0
	0.5	95.10	(2.73, 1.81)	0.60	0.62	95.15	(2.71, 1.77)	0.60	0.63	99.79	(0.06, 0.15)	0.29	0
	1	95.00	(1.93, 2.82)	0.41	0.38	95.08	(1.95, 2.73)	0.42	0.41	97.44	(0, 2.56)	0	0
	1.5	95.08	(1.30, 3.49)	0.27	0.65	95.25	(1.33, 3.29)	0.29	0.69	92.68	(0, 7.32)	0	0
	2	94.57	(0.52, 4.91)	0.10	0.97	94.87	(0.51, 4.62)	0.10	1.02	89.15	(0, 10.85)	0	0

Table S2 Estimated CP (%), ML (%), MR (%), ML/(ML+MR) and DP (%) of the two-sided 95% CI when $N = 500$, $\rho = 0.05$, and $\lambda_2 = 2$ for the LR, Fieller's and delta methods

		LR				Fieller				Delta			
p	γ	CP	(ML, MR)	$\frac{ML}{ML+MR}$	DP	CP	(ML, MR)	$\frac{ML}{ML+MR}$	DP	CP	(ML, MR)	$\frac{ML}{ML+MR}$	DP
0.1	0	94.89	(4.08, 1.03)	0.80	1.64	94.99	(4.12, 0.89)	0.82	2.23	100	(0, 0)	—	0
	0.5	94.68	(2.16, 2.56)	0.46	0.89	95.41	(2.20, 1.73)	0.56	1.01	95.62	(0, 4.38)	0	0
	1	94.95	(1.80, 3.03)	0.37	0.72	96.64	(1.80, 1.26)	0.59	0.82	85.67	(0, 14.33)	0	0
	1.5	95.06	(1.16, 3.74)	0.24	0.63	97.26	(1.08, 1.63)	0.40	0.66	82.23	(0, 17.77)	0	0
	2	95.03	(0.02, 4.95)	0	0.37	97.71	(0.02, 2.27)	0.01	0.38	80.92	(0, 19.08)	0	0
0.3	0	95.25	(2.62, 2.13)	0.55	0.40	95.27	(2.61, 2.12)	0.55	0.46	98.04	(1.94, 0.02)	0.99	0
	0.5	95.31	(2.57, 2.08)	0.55	0.14	95.34	(2.55, 2.07)	0.55	0.15	99.35	(0.20, 0.45)	0.31	0
	1	94.88	(2.37, 2.72)	0.47	0.06	94.97	(2.36, 2.64)	0.47	0.07	96.42	(0, 3.58)	0	0
	1.5	94.79	(2.22, 2.96)	0.43	0.21	95.04	(2.23, 2.71)	0.45	0.21	93.33	(0, 6.67)	0	0
	2	94.96	(1.94, 3.10)	0.38	0.06	95.14	(1.91, 2.95)	0.39	0.05	91.77	(0, 8.23)	0	0

Table S3 Estimated CP (%), ML (%), MR (%), ML/(ML+MR) and DP (%) of the two-sided 95% CI when $N = 2000$, $\rho = 0.05$, and $\lambda_2 = 1.5$ for the LR, Fieller's and delta methods

		LR				Fieller				Delta			
p	γ	CP	(ML, MR)	$\frac{\text{ML}}{\text{ML+MR}}$	DP	CP	(ML, MR)	$\frac{\text{ML}}{\text{ML+MR}}$	DP	CP	(ML, MR)	$\frac{\text{ML}}{\text{ML+MR}}$	DP
0.1	0	94.89	(4.03, 1.08)	0.79	1.53	94.93	(4.01, 1.06)	0.79	1.63	99.92	(0.08, 0)	1	0
	0.5	95.20	(1.93, 2.43)	0.44	0.63	95.29	(1.95, 2.28)	0.46	0.67	95.47	(0, 4.53)	0	0
	1	94.67	(1.84, 3.30)	0.36	0.66	95.18	(1.84, 2.81)	0.40	0.64	87.11	(0, 12.89)	0	0
	1.5	94.94	(1.23, 3.78)	0.25	0.38	95.57	(1.23, 3.15)	0.28	0.42	85.08	(0, 14.92)	0	0
	2	94.50	(0.03, 5.47)	0.01	0.15	95.27	(0.03, 4.70)	0.01	0.16	83.80	(0, 16.20)	0	0
0.3	0	95.11	(2.38, 2.51)	0.49	0.34	95.13	(2.38, 2.49)	0.49	0.35	97.85	(2.11, 0.04)	0.98	0
	0.5	94.60	(2.58, 2.80)	0.48	0.06	94.61	(2.58, 2.79)	0.48	0.05	98.76	(0.28, 0.96)	0.23	0
	1	94.88	(2.51, 2.60)	0.49	0.02	94.91	(2.52, 2.56)	0.50	0.02	96.25	(0, 3.75)	0	0
	1.5	94.86	(2.40, 2.73)	0.47	0.05	94.98	(2.40, 2.61)	0.48	0.05	93.50	(0, 6.50)	0	0
	2	94.75	(2.23, 3.02)	0.42	0.01	94.86	(2.22, 2.92)	0.43	0.01	92.43	(0, 7.57)	0	0

Table S4 Estimated CP (%), ML (%), MR (%), ML/(ML+MR) and DP (%) of the two-sided 95% CI when $N = 2000$, $\rho = 0.05$, and $\lambda_2 = 2$ for the LR, Fieller's and delta methods

		LR				Fieller				Delta			
p	γ	CP	(ML, MR)	$\frac{ML}{ML+MR}$	DP	CP	(ML, MR)	$\frac{ML}{ML+MR}$	DP	CP	(ML, MR)	$\frac{ML}{ML+MR}$	DP
0.1	0	95.17	(2.77, 2.06)	0.57	1.10	95.21	(2.78, 2.01)	0.58	1.25	99.62	(0.31, 0.07)	0.82	0
	0.5	95.06	(2.54, 2.34)	0.52	0.13	95.17	(2.58, 2.17)	0.54	0.13	95.30	(0, 4.70)	0	0
	1	94.51	(2.30, 3.19)	0.42	0.06	95.01	(2.35, 2.64)	0.47	0.06	90.40	(0, 9.60)	0	0
	1.5	94.82	(2.01, 3.17)	0.39	0	95.34	(2.05, 2.61)	0.44	0	89.82	(0, 10.18)	0	0
	2	94.80	(1.21, 3.99)	0.23	0	95.42	(1.26, 3.32)	0.28	0	89.16	(0, 10.84)	0	0
0.3	0	94.93	(2.54, 2.53)	0.50	0	94.97	(2.52, 2.51)	0.50	0	96.52	(3.09, 0.39)	0.89	0
	0.5	95.07	(2.45, 2.48)	0.50	0	95.07	(2.45, 2.48)	0.50	0	96.93	(1.28, 1.79)	0.42	0
	1	95.12	(2.29, 2.59)	0.47	0	95.13	(2.32, 2.55)	0.48	0	95.97	(0.19, 3.84)	0.05	0
	1.5	95.06	(2.45, 2.49)	0.50	0	95.14	(2.47, 2.39)	0.51	0	95.01	(0.01, 4.98)	0	0
	2	95.03	(2.50, 2.47)	0.50	0	95.09	(2.53, 2.38)	0.52	0	94.55	(0, 5.45)	0	0

Table S5 Estimated size (%) for testing $H_0: \ \gamma = \gamma_0$ with $N = 500$ and $\rho = 0$ for the LR, Fieller's and delta methods

		$\lambda_2 = 1.5$			$\lambda_2 = 2$		
p	γ_0	LR	Fieller	Delta	LR	Fieller	Delta
0.1	0	5.25	5.07	0	5.41	5.22	0
	0.5	4.22	3.19	4.28	4.76	3.80	5.56
	1	3.87	1.82	**20.62**	3.93	2.22	**15.95**
	1.5	3.61	1.28	**29.17**	3.44	1.63	**18.92**
	2	3.32	1.01	**32.29**	3.51	1.63	**20.29**
0.3	0	5.28	5.25	0.78	5.12	5.07	1.90
	0.5	4.79	4.75	0.25	4.99	4.99	0.87
	1	5.23	5.12	3.30	4.85	4.72	3.63
	1.5	4.78	4.61	**7.71**	5.19	4.97	**7.49**
	2	5.28	4.84	**11.89**	5.12	4.82	**8.20**

Table S6 Estimated size (%) for testing $H_0: \gamma = \gamma_0$ with $N = 2000$ and $\rho = 0$ for the LR, Fieller's and delta methods

		$\lambda_2 = 1.5$			$\lambda_2 = 2$		
p	γ_0	LR	Fieller	Delta	LR	Fieller	Delta
0.1	0	5.11	5.09	0.02	4.55	4.52	0.19
	0.5	5.62	5.22	**6.71**	5.08	4.73	**7.37**
	1	4.91	4.12	**15.84**	5.61	4.69	**11.13**
	1.5	5.51	4.41	**18.82**	5.23	4.23	**12.08**
	2	5.32	4.19	**18.99**	5.55	4.43	**12.68**
0.3	0	4.77	4.77	2.11	4.98	4.97	3.36
	0.5	5.00	4.99	0.92	5.03	5.03	3.06
	1	5.20	5.14	4.09	4.87	4.83	3.95
	1.5	4.95	4.90	**6.42**	5.32	5.24	5.35
	2	5.42	5.32	**8.14**	5.14	5.11	**6.19**

Table S7 Estimated size (%) for testing $H_0: \ \gamma = \gamma_0$ with $N = 500$ and $\rho = 0.05$ for the LR, Fieller's and delta methods

		$\lambda_2 = 1.5$			$\lambda_2 = 2$		
p	γ_0	LR	Fieller	Delta	LR	Fieller	Delta
0.1	0	5.07	4.88	0	5.11	5.01	0
	0.5	5.37	4.63	4.27	5.32	4.59	4.38
	1	5.08	2.96	**17.38**	5.05	3.36	**14.33**
	1.5	4.81	2.34	**24.77**	4.94	2.74	**17.77**
	2	5.15	2.31	**29.10**	4.97	2.29	**19.08**
0.3	0	5.01	4.95	0.87	4.75	4.73	1.96
	0.5	4.90	4.85	0.21	4.69	4.66	0.65
	1	5.00	4.92	2.56	5.12	5.03	3.58
	1.5	4.92	4.75	**7.32**	5.21	4.96	**6.67**
	2	5.43	5.13	**10.85**	5.04	4.86	**8.23**

Table S8 Estimated size (%) for testing $H_0: \gamma = \gamma_0$ with $N = 2000$ and $\rho = 0.05$ for the LR, Fieller's and delta methods

		$\lambda_2 = 1.5$			$\lambda_2 = 2$		
p	γ_0	LR	Fieller	Delta	LR	Fieller	Delta
0.1	0	5.11	5.07	0.08	4.83	4.79	0.38
	0.5	4.80	4.71	4.53	4.94	4.83	4.70
	1	5.33	4.82	**12.89**	5.49	4.99	**9.60**
	1.5	5.06	4.43	**14.92**	5.18	4.66	**10.18**
	2	5.50	4.73	**16.20**	5.20	4.58	**10.84**
0.3	0	4.89	4.87	2.15	5.07	5.03	3.48
	0.5	5.40	5.39	1.24	4.93	4.93	3.07
	1	5.12	5.09	3.75	4.88	4.87	4.03
	1.5	5.14	5.02	**6.50**	4.94	4.86	4.99
	2	5.25	5.14	**7.57**	4.97	4.91	5.45

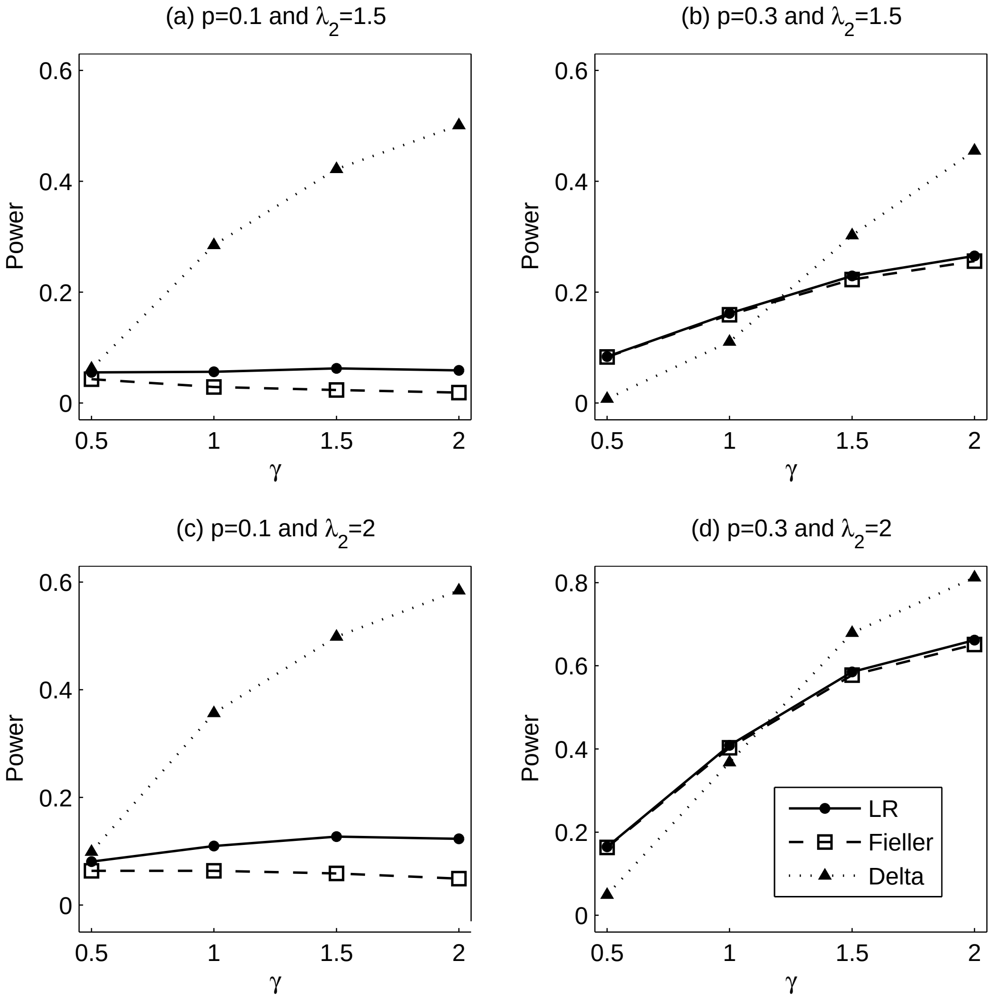

Fig. S1 Power comparison of the LR, Fieller's and delta methods against γ. The simulation is based on 10,000 replicates with $N = 500$, $\rho = 0$ and $\gamma_0 = 0$.

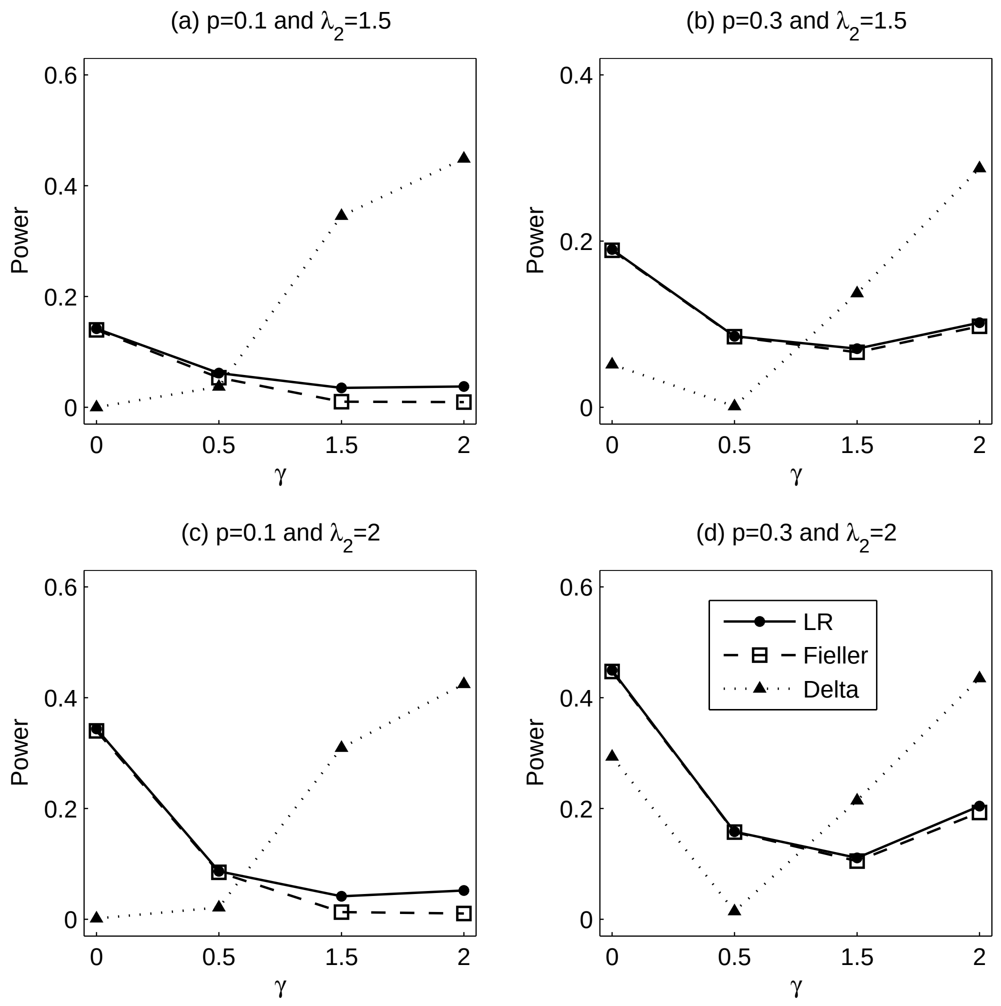

Fig. S2 Power comparison of the LR, Fieller's and delta methods against γ. The simulation is based on 10,000 replicates with $N = 500$, $\rho = 0$ and $\gamma_0 = 1$.

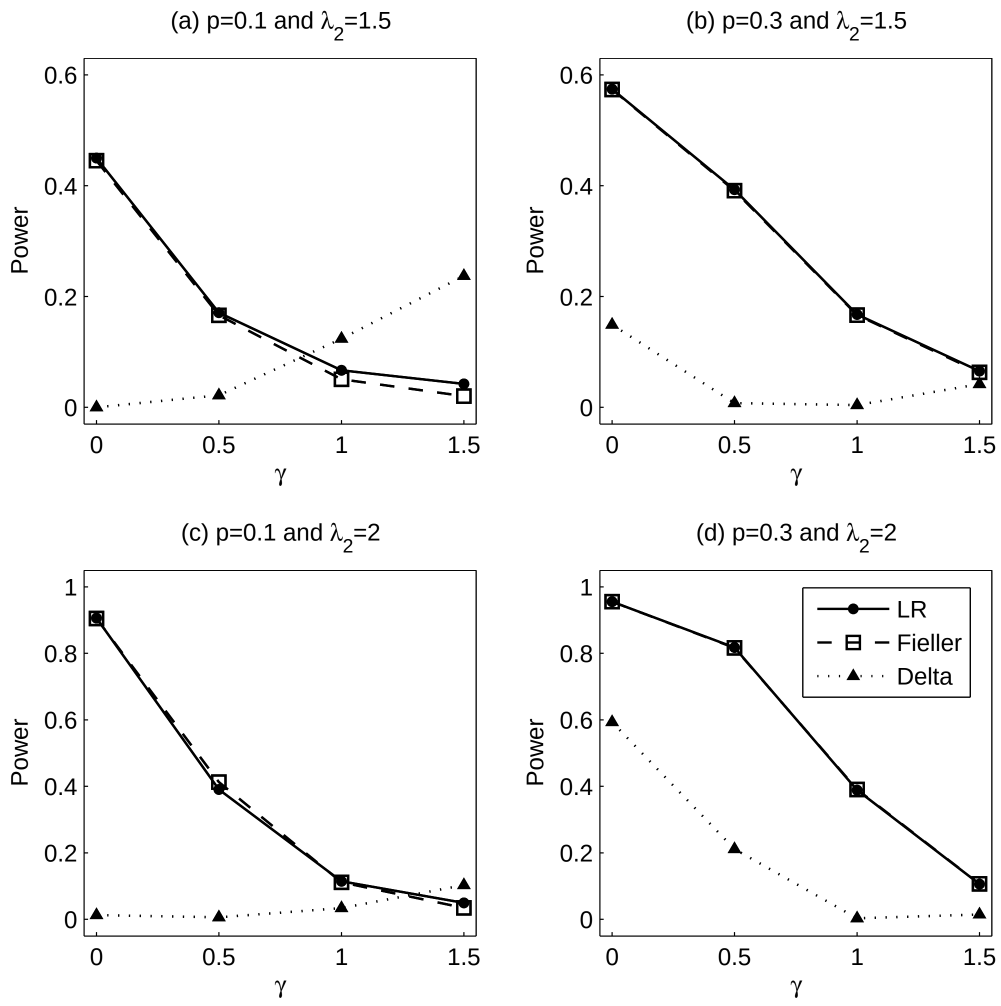

Fig. S3 Power comparison of the LR, Fieller's and delta methods against γ. The simulation is based on 10,000 replicates with $N = 500$, $\rho = 0$ and $\gamma_0 = 2$.

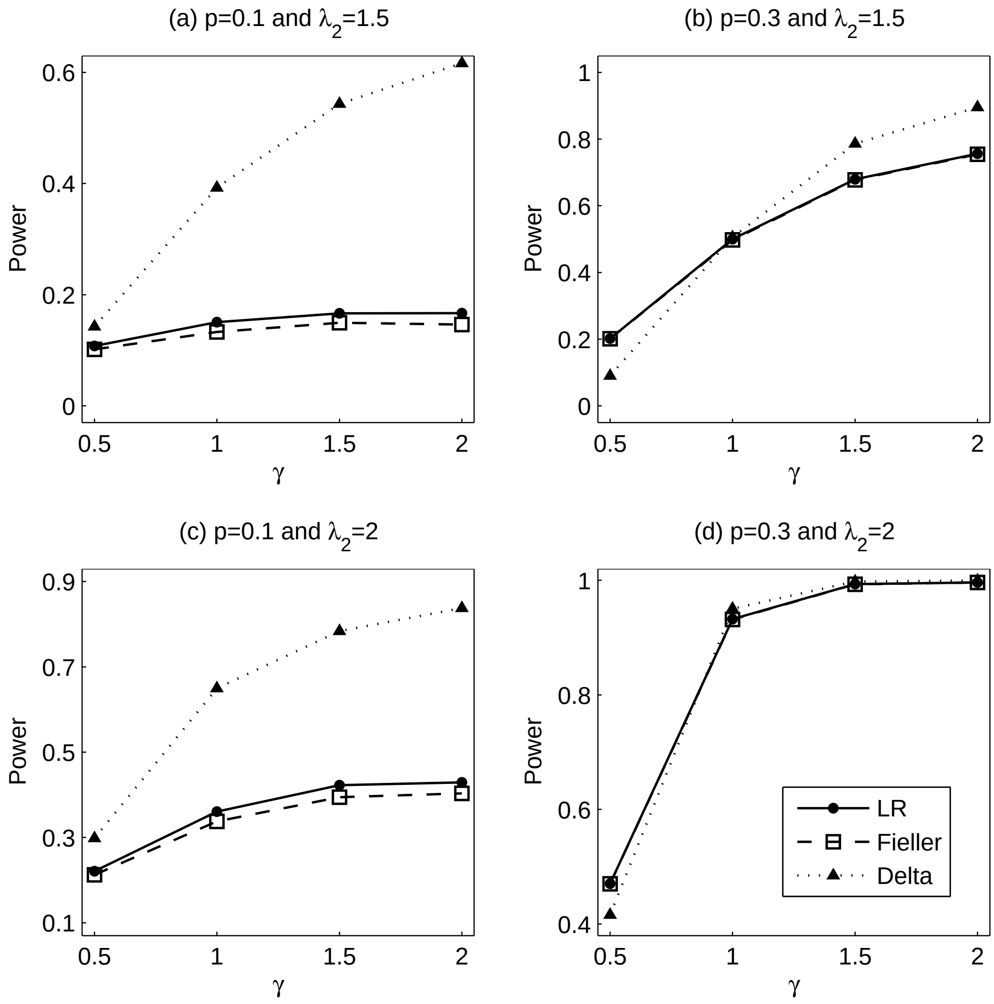

Fig. S4 Power comparison of the LR, Fieller's and delta methods against γ. The simulation is based on 10,000 replicates with $N = 2000$, $\rho = 0$ and $\gamma_0 = 0$.

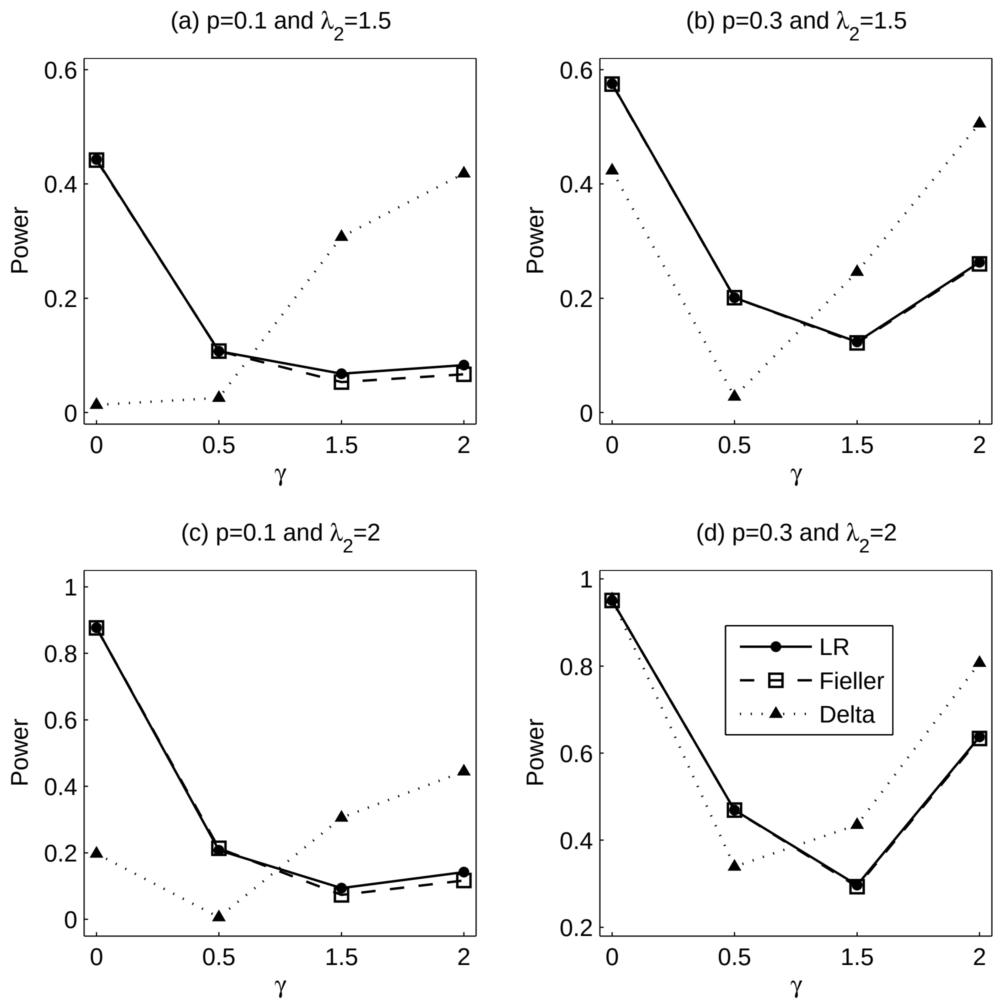

Fig. S5 Power comparison of the LR, Fieller's and delta methods against γ. The simulation is based on 10,000 replicates with $N = 2000$, $\rho = 0$ and $\gamma_0 = 1$.

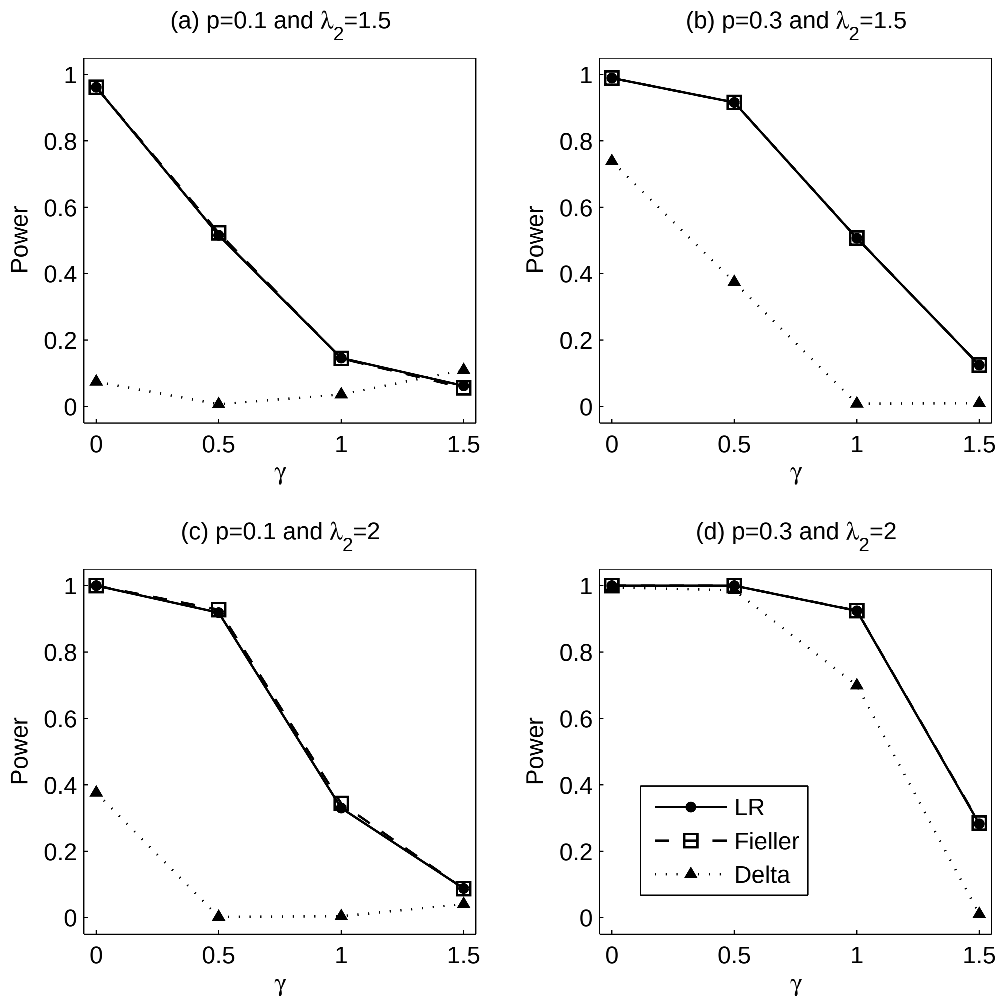

Fig. S6 Power comparison of the LR, Fieller's and delta methods against γ. The simulation is based on 10,000 replicates with $N = 2000$, $\rho = 0$ and $\gamma_0 = 2$.

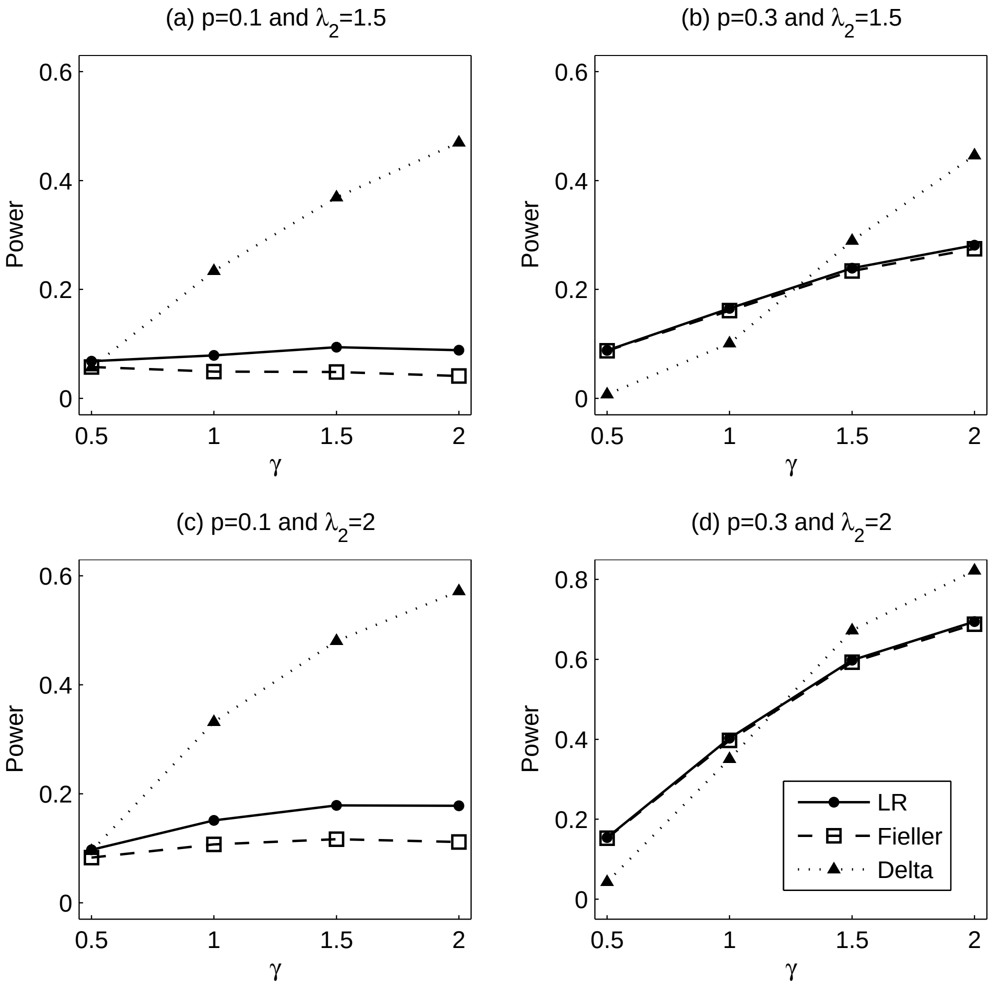

Fig. S7 Power comparison of the LR, Fieller's and delta methods against γ. The simulation is based on 10,000 replicates with $N = 500$, $\rho = 0.05$ and $\gamma_0 = 0$.

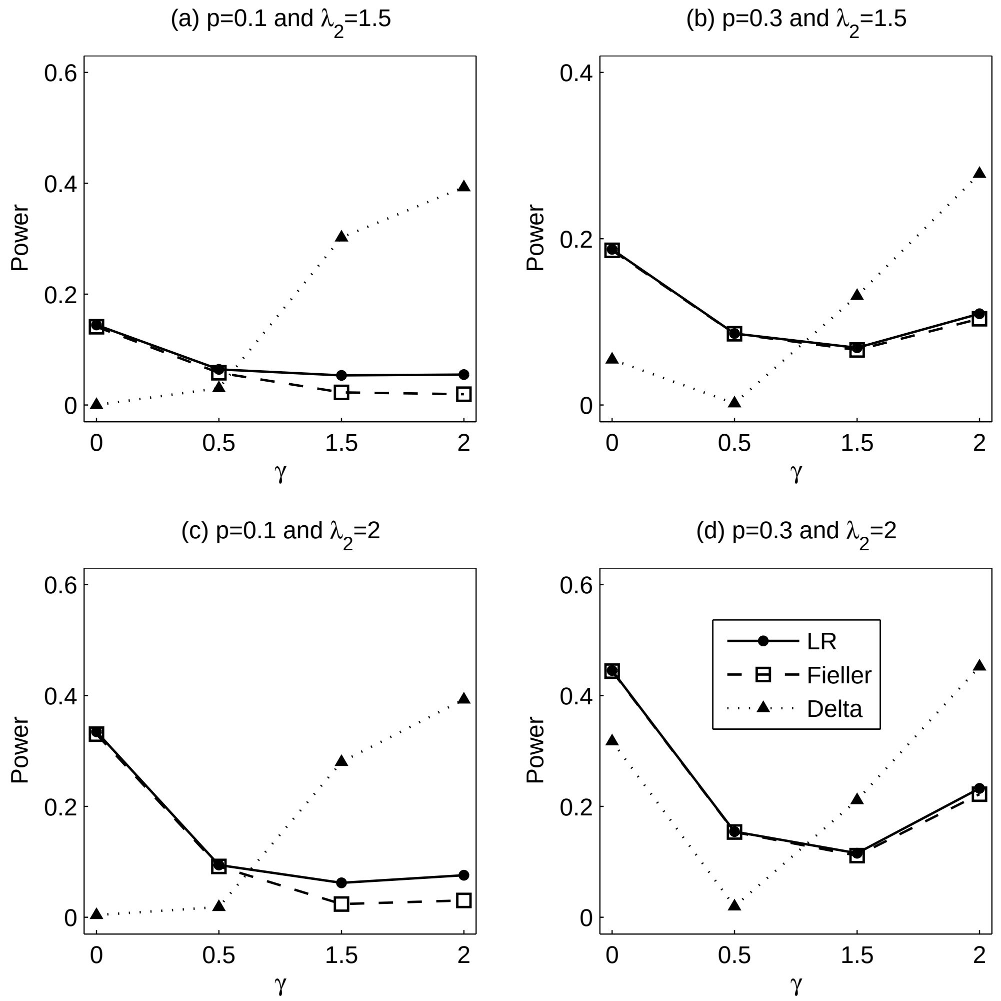

Fig. S8 Power comparison of the LR, Fieller's and delta methods against γ. The simulation is based on 10,000 replicates with $N = 500$, $\rho = 0.05$ and $\gamma_0 = 1$.

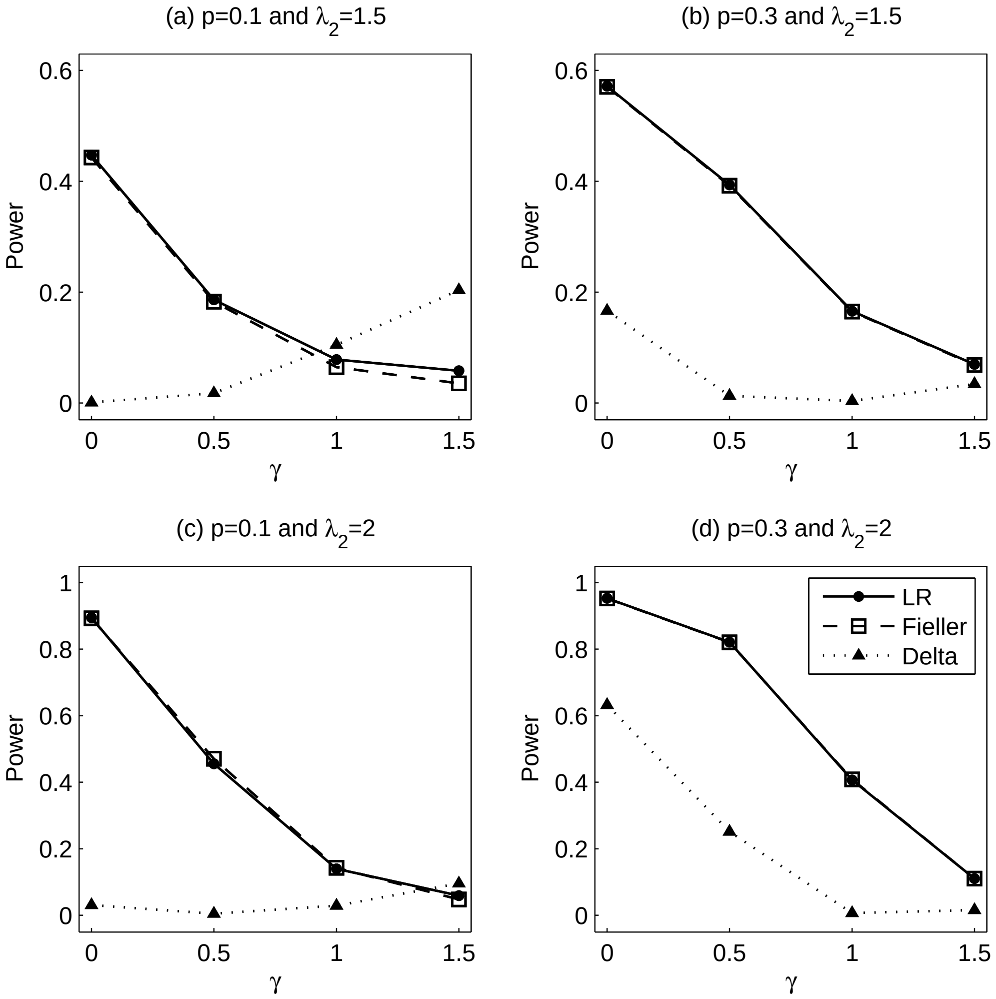

Fig. S9 Power comparison of the LR, Fieller's and delta methods against γ. The simulation is based on 10,000 replicates with $N = 500$, $\rho = 0.05$ and $\gamma_0 = 2$.

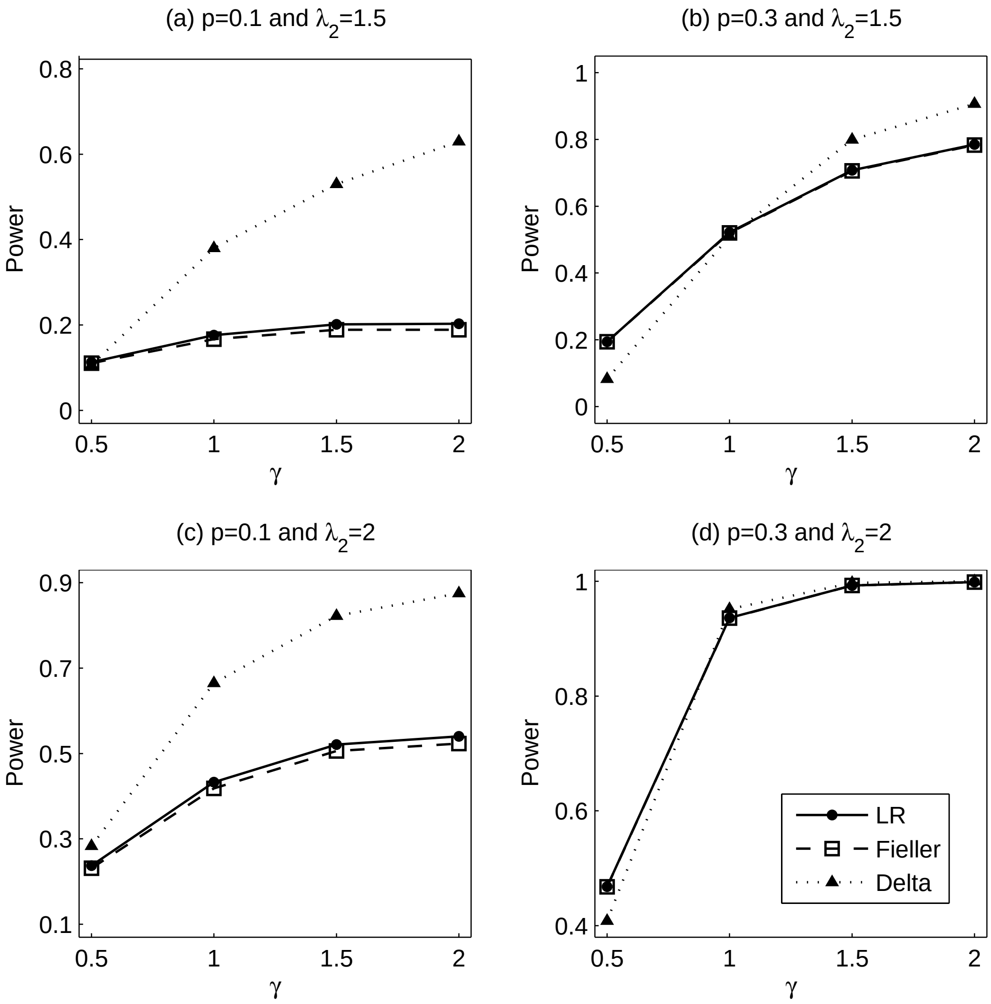

Fig. S10 Power comparison of the LR, Fieller's and delta methods against γ. The simulation is based on 10,000 replicates with $N = 2000$, $\rho = 0.05$ and $\gamma_0 = 0$.

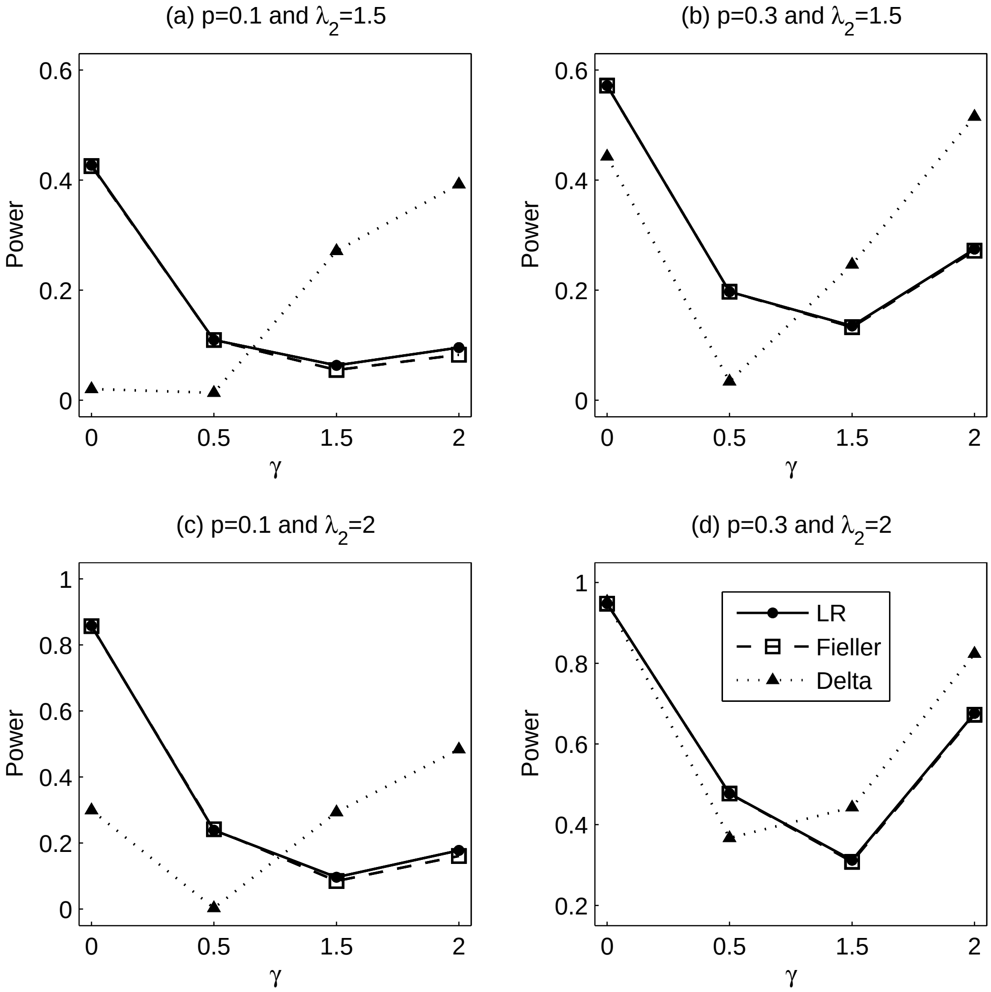

Fig. S11 Power comparison of the LR, Fieller's and delta methods against γ. The simulation is based on 10,000 replicates with $N = 2000$, $\rho = 0.05$ and $\gamma_0 = 1$.

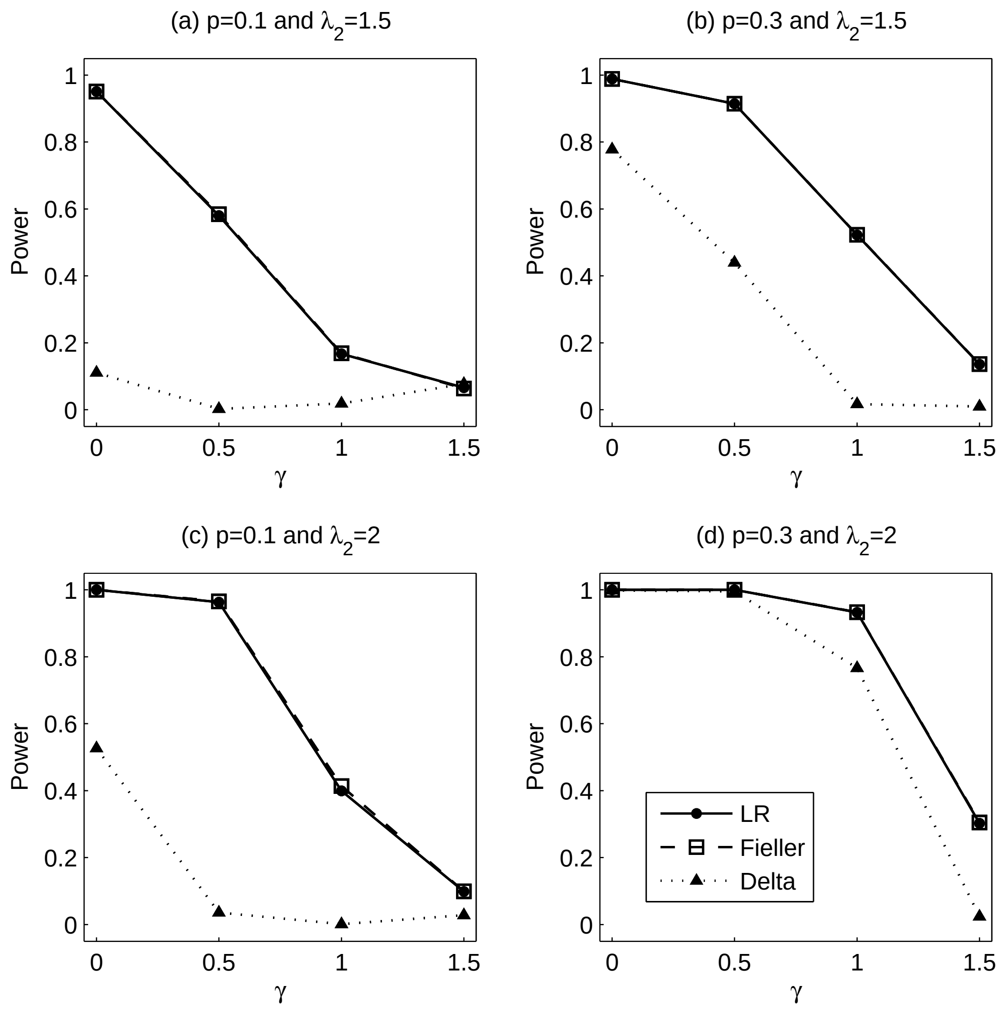

Fig. S12 Power comparison of the LR, Fieller's and delta methods against γ. The simulation is based on 10,000 replicates with $N = 2000$, $\rho = 0.05$ and $\gamma_0 = 2$.
